# Vertex‐Shared Linear Superatomic Molecules: Stepping Stones to Novel Materials Composed of Noble Metal Clusters

**DOI:** 10.1002/smsc.202300024

**Published:** 2023-04-24

**Authors:** Yoshiki Niihori, Sayuri Miyajima, Ayaka Ikeda, Taiga Kosaka, Yuichi Negishi

**Affiliations:** ^1^ Research Institute for Science and Technology Tokyo University of Science Kagurazaka Shinjuku-ku Tokyo 162–8601 Japan; ^2^ Department of Applied Chemistry Faculty of Science Tokyo University of Science Kagurazaka Shinjuku-ku Tokyo 162–8601 Japan

**Keywords:** connections, geometrical structures, ligand-protected metal clusters, superatomic molecules

## Abstract

Extremely small metal clusters composed of noble metal atoms (M) have orbitals similar to those of atoms and therefore can be thought of as artificial atoms or superatoms. If these superatoms can be assembled into molecular analogs, it might be possible to create materials with new characteristics and properties that are different from those of existing substances. Therefore, the concept of superatomic molecules has attracted significant attention. The present review focuses on vertex‐shared linear M_12*n*+1_ superatomic molecules formed via the sharing of a single metal atom between M_13_ superatoms having icosahedral cores and summarizes the knowledge obtained to date in this regard. This summary discusses the most suitable ligand combinations for the synthesis of M_12*n*+1_ superatomic molecules along with the valence electron numbers, stability, optical absorption characteristics, and luminescence properties of the M_12*n*+1_ superatomic molecules fabricated to date. This information is expected to assist in the production of many M_12*n*+1_ superatomic molecules with novel structures and physicochemical properties in the future.

## Graphical abstract

Superatomic molecules are synthesized by assembling icosahedral superatoms with electronic structures similar to atoms and are summarized according to their number of linkages and coordination environment. These superatomic molecules have various physicochemical properties not found in simple molecules composed of atoms, especially high stability and structure‐dependent optical properties, and are promising as next‐generation functional nanomaterials.

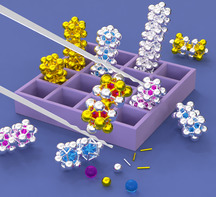

## 1 Introduction

**Figure 1:**
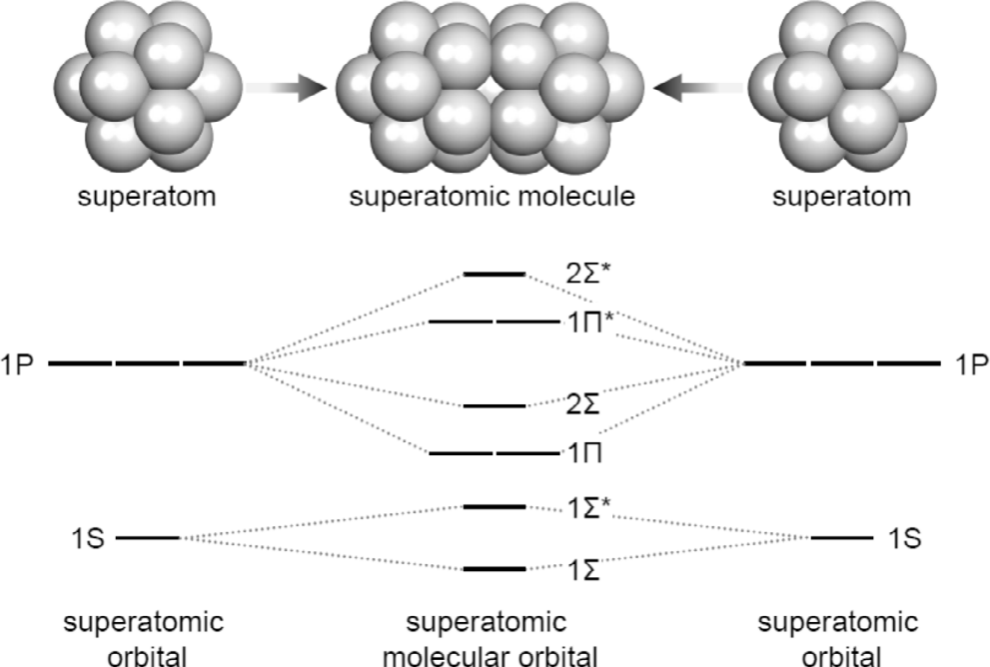
Diagram showing the formation of a superatomic molecule and the associated orbitals.^[3b]^

Metal clusters, which can be represented as M_*n*_ where M is gold (Au), silver (Ag), copper (Cu), or other metals, and *n* is the number of metal atoms in the cluster, have unique electron orbitals. In these M_*n*_ clusters, the valence electrons can be regarded as being confined in spherically symmetrical regions within a positive charge potential field (representing the so‐called jellium model^[^ ^1^ ^]^). The orbitals (S, P, D, F, etc.) in this model are analogs to those of the corresponding atomic orbitals (s, p, d, f, etc.).^[^ ^2^ ^]^ For these reasons, M_*n*_ clusters are sometimes referred to as superatoms (**Figure** **1**).^[^ ^3^ ^]^ Examples include [Au_13_(PMe_2_Ph)]_10_Cl_2_]^3+^ (Pme_2_Ph = dimethylphenylphosphine, Cl = chloride),^[^ ^4^ ^]^ [Au_13_(dppe)_5_Cl_2_]^3+^ (dppe = bis(diphenylphosphino)ethane),^[^ ^5^ ^]^ [Au_9_M_4_(PmePh_2_)_8_Cl_4_]^+^ (M = Au, Ag, Cu; PmePh_2_ = methyldiphenylphosphine),^[^ ^6^ ^]^ [Au_12_Pd(dppe)(PPh_3_)_6_Cl_4_]^0^ (Pd = palladium, PPh_3_ = triphenylphosphine),^[^ ^7^ ^]^ and [Ag_12_Pt(dppm)_5_(DMBT)_2_]^2+^ (Pt = platinum, dppm = bis(diphenylphosphino)methane; DMBT = 2,4‐dimethylbenzenethiolate),^[^ ^8^ ^]^ in which an icosahedral M_13_ core is surrounded by ligands. The formation of molecular analogs from such superatoms could potentially create materials with new properties and functions different from those of existing substances (Figure 1).^[3a]^

**Figure 2:**
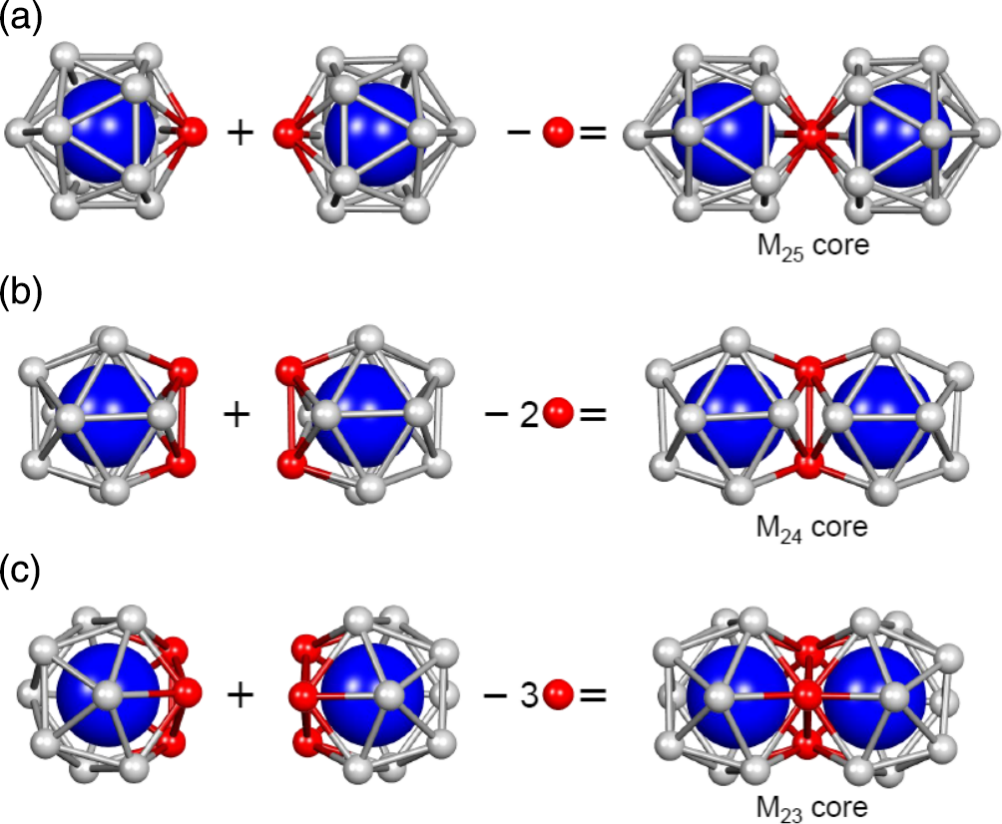
a–c) Diagrams of vertex‐sharing (a), edge‐sharing (b), and face‐sharing (c) superatomic molecules.

Several methods describing the connection of superatoms have been reported. Among these techniques, the connection of superatoms via shared metal atoms is of interest in that this process promotes the mixing of orbitals between superatoms. As such, electronic structures that are different from those of each individual superatom can be obtained.[^3^, ^9^] In the case of M_13_ clusters, the superatoms can be connected by sharing one (via a vertex; **Figure** **2**a),^[^ ^10^ ^]^ two (via an edge; Figure 2b), or three (via a face; Figure 2c)^[^ ^11^ ^]^ atoms. These processes create superatomic molecules consisting of 2 × 13 − 1 = 25 atoms, 2 × 13 − 2 = 24 atoms, or 2 × 13 − 3 = 23 atoms, respectively (Figure 2).

This review summarizes the state‐of‐the‐art concerning research into vertex‐sharing linear M_12*n*+1_ superatomic molecules (*n* ≥ 2) formed by the sharing of a single metal atom, which has been the most studied of these superatomic molecules. This article presents the latest knowledge regarding such molecules and describes the novel structures and physicochemical properties that can be obtained.

## 2 M_12*n*+1_ Superatomic Molecules (*n* ≥ 2)

**Figure 3:**
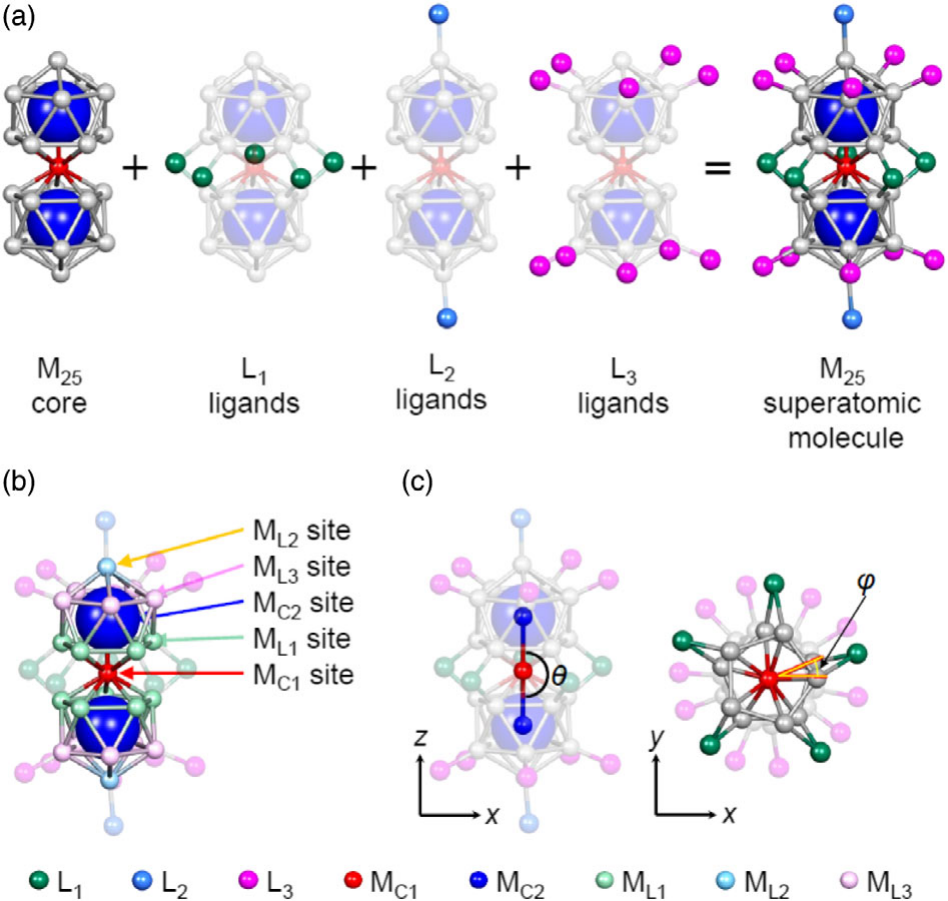
a) The anatomy of an M_25_ superatomic molecule comprising an M_25_ core and three types of ligands. b) The five different metal sites in an M_25_ core. c) Characteristic geometrical parameters of an M_25_ superatomic molecule. In these figures, carbon and hydrogen atoms in the phosphine ligands are omitted for clarity.

**Figure 4:**
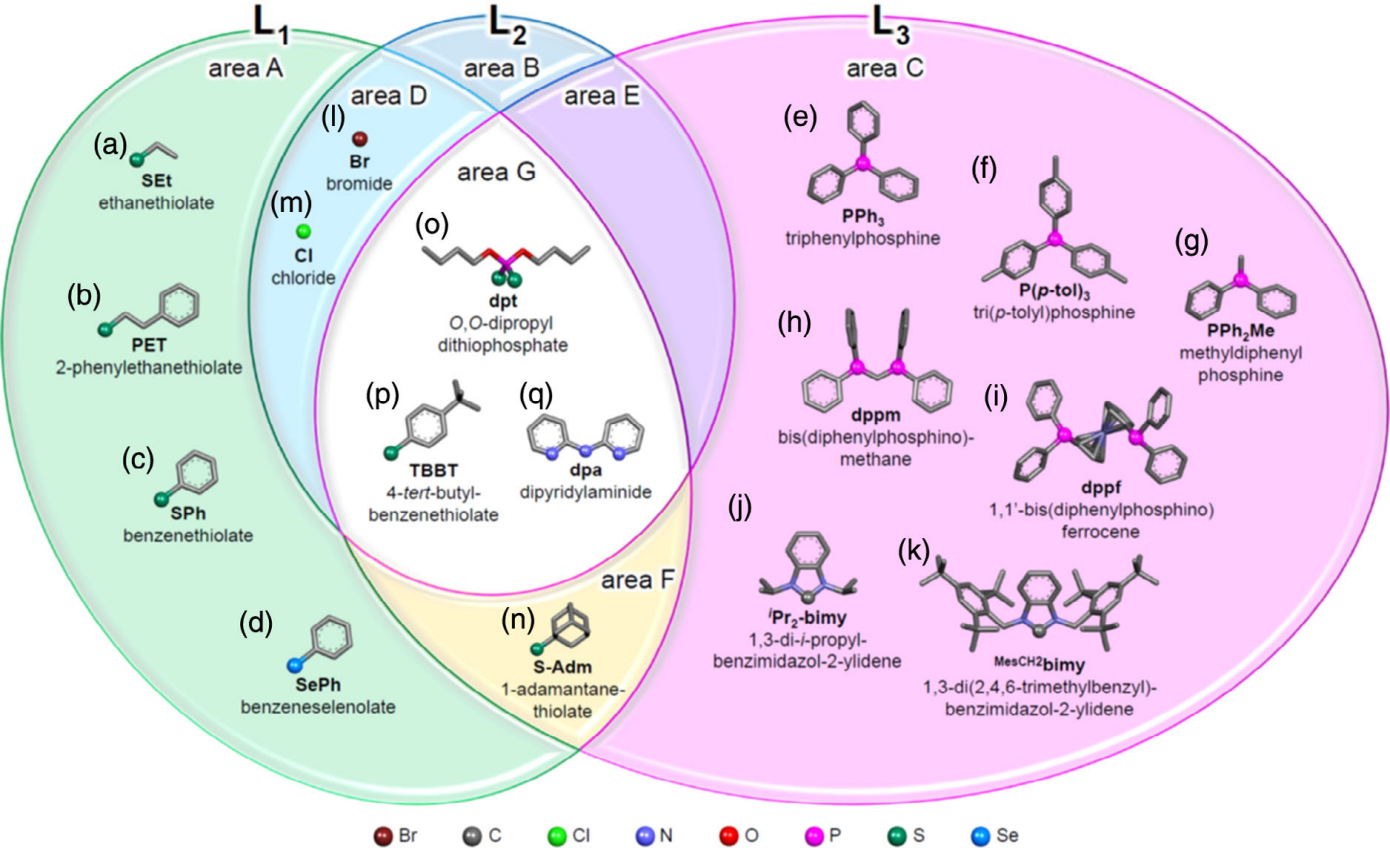
Classification of ligands used for the formation of superatomic molecules based on a Venn diagram. L_1_, L_2_, and L_3_ represent characteristic ligand sites (see also Figure 3). a) Ethanethiolate, b) 2‐phenylethanethiolate, c) benzenethiolate, d) benzeneselenolate, e) triphenylphosphine, f) tri(*p*‐tolyl)phosphine, g) methyldiphenylphosphine, h) bis(diphenylphosphino)methane, i) 1,1′‐bis(diphenylphosphino)ferrocene, j) 1,3‐di‐*i*‐propylbenzimidazol‐2‐ylidene, k) 1,3‐di(2,4,6‐trimethylbenzyl)benzimidazol‐2‐ylidene, l) bromide, m) chloride, n) 1‐adamantanethiolate, o) *O*,*O*‐dipropyldithiophosphate, p) 4‐*tert*‐buthylbenzenethiolate, and q) dipyridylaminide ligands.

The basic geometry of an M_25_ superatomic molecule (*n* = 2) is shown in **Figure** **3**. The M_25_ core is formed by two icosahedral M_13_ superatomic molecules sharing one metal atom and has a fivefold axis of symmetry in the long axis direction of the molecule. In this structure, more than five L_1_ ligands (Figure 3a) are coordinated to the metal atom at the M_L1_ site, two L_2_ ligands (Figure 3a) are coordinated to the metal atom at the M_L3_ site, and approximately 10 L_3_ ligands (Figure 3a) are coordinated to the metal atom at the M_L3_ site (Figure 3b). The ligands of superatomic molecules having M_12*n*+1_ cores (*n* ≥ 2) for which geometric structures have been determined are categorized in the Venn diagram,^[^ ^12^ ^]^ as presented in **Figure** **4**. In many cases, anionic ligands such as halogens (X) or chalcogenides (ER) are used as the L_1_ and L_2_ ligands (areas A, D, G, and F in Figure 4). The L_3_ ligands are typically neutral with an unshared electron pair, such as a phosphine (PR_3_) or N‐heterocyclic carbene (NHC) (area C in Figure 4). The coordination styles of the ligands are basically the same in linear M_37_ (*n* = 3) and M_49_ (*n* = 4) superatomic molecules as M_25_ superatomic molecules. The Venn diagram presented here demonstrates that there are currently no ligands that coordinate only to the L_2_ site (area B in Figure 4) or to both L_2_ and L_3_ sites (area E in Figure 4). Because there is a wide range of other ligands that could potentially be used to produce ligand‐protected metal clusters,^[^ ^13^ ^]^ including acetylide (C ≡ CR),^[^ ^14^ ^]^ arsine (AsR_3_),^[^ ^15^ ^]^ and tellurolate (TeR) moieties,^[^ ^16^ ^]^ it is expected that ligands satisfying these conditions will be discovered in the future.

The number of valence electrons (n*) in these superatomic molecules can be estimated using a method based on the jellium model proposed by Mingos et al.^[^ ^17^ ^]^ or the superatom model more recently developed by Häkkinen et al.^[^ ^18^ ^]^ and Cheng and Yang et al.^[^ ^19^ ^]^ **Table** **1** summarizes the chemical compositions, geometric structure parameters, and numbers of valence electrons for representative superatomic molecules.^[^ ^10^ ^]^ Here, the torsion angle (*φ*, the average of five dihedral angles) and the bond angle (*θ*) between the two M_13_ cores (see Figure 3c) are also provided in addition to the symmetry of the structure and the ligands. The following text discusses the knowledge obtained to date while classifying superatomic molecules according to the type of ligand incorporated.

**Table 1:** Chemical compositions, core metals, molecular symmetries, core symmetries, torsion, and bond angles between the superatoms, L_1_, L_2_, and L_3_ ligands, charge states, and numbers of valence electrons of representative vertex‐shared linear superatomic molecules

Entry	# of M_13_	Chemical compositiona)	Core metal	Mol. sym.b), c)	Core sym.c)	*φ*°d)	*θ*°e)	L_1_ ligands	L_2_ ligands	L_3_ ligands	*z* f)	n*g)	Reference
1	2	[Ag_12_Au_13_(P(*p*‐tol)_3_)_10_Cl_7_](SbF_6_)_2_	Ag_12_Au_13_	*D*_5_	*D*_5_	10	176	5(μ‐Cl)	2Cl	10P	+2	16	[10a]
2	2	[Ag_12_Au_13_(PPh_3_)_10_Br_8_](Br)	Ag_12_Au_13_	*C*_2h_	*D*_5d_	36	175	4(μ‐Br) + 2(μ^3^‐Br)	2Br	10P	+1	16	[10b]
3	2	[Ag_12_Au_13_(PPh_3_)_10_Br_8_](SbF_6_)	Ag_12_Au_13_	*C*_s_	*D*_5h_	0	177	5(μ‐Br) + (μ^4^‐Br)	2Br	10P	+1	16	[10c]
4	2	[Ag_12_Au_13_(P(*p*‐tol)_3_)_10_Br_8_](PF_6_)	Ag_12_Au_13_	*C*_2h_	*D*_5d_	36	180	2(μ‐Br) + 4(μ^3^‐Br)	2Br	10P	+1	16	[10d]
5	2	[Ag_12_Au_13_(PPh_3_)_10_Cl_8_](SbF_6_)	Ag_12_Au_13_	*C*_2v_	*D*_5h_	0	178	5(μ‐Cl) + (μ^4^‐Cl)	2Cl	10P	+1	16	[10e,f]
6	2	[Ag_12_Au_13_(PPh_3_)_10_Cl_8_](SbF_6_)	Ag_12_Au_13_	*C*_2_	*D*_5(d)_	33	176	4(μ‐Cl) + 2(μ^3^‐Cl)	2Cl	10P	+1	16	[10f]
7	2	[Ag_12_Au_13_(P(*p*‐tol)_3_)_10_Cl_8_](PF_6_)	Ag_12_Au_13_	*C*_2_	*D*_5_	17	177	4(μ‐Cl) + 2(μ^3^‐Cl)	2Cl	10P	+1	16	[10g]
8	2	[Ag_13−*X*_Au_13+*X*_(PPh_3_)_10_Cl_8_](Cl)_2_	Ag_13−*X*_Au_13+*X*_	*C*_2_	*D*_5(d)_	31	176	4(μ‐Cl) + 2(μ^3^‐Cl)	2Cl	10P	+2	15	[10h]
9	2	[Ag_13_Au_12_(PPh_2_Me)_10_Br_9_]^0^	Ag_13_Au_12_	*C*_(s)_	*D*_5(d)_	34	177	4(μ‐Br) + 3(μ^3^‐Br)	2Br	10P	0	15	[10i]
10	2	[Ag_17_Au_8_(PPh_3_)_10_Cl_10_]^0^	Ag_17_Au_8_	*C*_2_	*C*_2(h)_	33	173	2Cl + 4(μ‐Cl) + 2(μ^3^‐Cl)	2Cl	10P	0	15	[10j]
11	2	[Ag_23_Pd_2_(PPh_3_)_10_Br_7_]^0^	Ag_23_Pd_2_	*D*_5_	*D*_5_	11	178	5(μ‐Br)	2Br	10P	0	16	[10k]
12	2	[Ag_23_Pd_2_(PPh_3_)_10_Cl_7_]^0^	Ag_23_Pd_2_	*D*_5h_	*D*_5h_	0	177	5(μ‐Cl)	2Cl	10P	0	16	[10l]
13	2	[Ag_23_Pt_2_(PPh_3_)_10_Br_7_]^0^	Ag_23_Pt_2_	*D*_5_	*D*_5_	10	178	5(μ‐Br)	2Br	10P	0	16	[10k]
14	2	[Ag_23_Pt_2_(PPh_3_)_10_Cl_7_]^0^	Ag_23_Pt_2_	*D*_5h_	*D*_5h_	0	178	5(μ‐Cl)	2Cl	10P	0	16	[10m]
15	2	[Au_23_Pd_2_(PPh_3_)_10_Br_7_]^0^	Au_23_Pd_2_	*D*_5h_	*D*_5h_	0	176	5(μ‐Br)	2Br	10P	0	16	[10n]
16	2	[Ag_12_Au_12_Ni(PPh_3_)_10_Cl_7_](SbF_6_)	Ag_12_Au_12_Ni	*C*_5v_	*C*_5v_	0	178	5(μ‐Cl)	2Cl	10P	+1	16	[10o]
17	2	[Ag_12_Au_12_Pt(PPh_3_)_10_Cl_7_](Cl)	Ag_12_Au_12_Pt	*C*_5v_	*C*_5v_	0	179	5(μ‐Cl)	2Cl	10P	+1	16	[10p]
18	2	[Ag_12_Au_11_Pt_2_(PPh_3_)_10_Cl_7_]^0^	Ag_12_Au_11_Pt_2_	*D*_5h_	*D*_5h_	0	178	5(μ‐Cl)	2Cl	10P	0	16	[10q]
19	2	[Ag_13_Au_10_Pt_2_(PPh_3_)_10_Cl_7_]^0^	Ag_13_Au_10_Pt_2_	*D*_5h_	*D*_5h_	0	178	5(μ‐Cl)	2Cl	10P	0	16	[10r]
20	2	[Au_25_(PPh_3_)_10_(SEt)_5_Cl_2_](SbF_6_)_2_	Au_25_	*D*_5(h)_	*D*_5(h)_	1	178	5(μ‐S)	2Cl	10P	+2	16	[10s]
21	2	[Au_25_(PPh_3_)_10_(PET)_5_Cl_2_](SbF_6_)_2_	Au_25_	*D*_5(h)_	*D*_5(h)_	0	179	5(μ‐S)	2Cl	10P	+2	16	[10t]
22	2	[Au_25_(PPh_3_)_10_(SPh)_5_Cl_2_](Cl)_2_	Au_25_	*D*_5(h)_	*D*_5(h)_	0	179	5(μ‐S)	2Cl	10P	+2	16	[10u]
23	2	[Ag_*X*_Au_25−*X*_(PPh_3_)_10_(PET)_5_X_2_](SbF_6_)_2_	Ag_*X*_Au_25−*X*_	*–*	*–*	–	–	5(μ‐Cl)	2Cl	10P	+2	16	[10v]
24		[Au_25−*X*_Cu_*X*_(PPh_3_)_10_(PET)_5_Cl_2_](SbF_6_)_2_	Au_25−*X*_Cu_*X*_	*–*	*–*	–	–	5(μ‐Cl)	2Cl	10P	+2	16	[10w]
25	2	[AgAu_24_(PPh_3_)_10_(PET)_5_Cl_2_](SbF_6_)_2_	AgAu_24_	*C*_5v_	*C*_5v_	0	180	5(μ‐Cl)	2Cl	10P	+2	16	[10x]
26	2	[Au_24_Cu(PPh_3_)_10_(PET)_5_Cl_2_](SbF_6_)_2_	Au_24_Cu	*C*_5v_ + *C*_s_ h)	*C*_5v_ + *C*_s_ h)	–	–	5(μ‐Cl)	2Cl	10P	+2	16	[10x]
27	2	[Au_24_Pd(PPh_3_)_10_(PET)_5_Cl_2_](Cl)	Au_24_Pd	*C*_5(v)_	*C*_5(v)_	2	179	5(μ‐S)	2Cl	10P	+1	16	[10y]
28	2	[Au_25_(PPh_3_)_10_(SePh)_5_Cl_2_](SbF_6_)	Au_25_	*D*_5h_	*D*_5h_	0	179	5(μ‐Se)	2Cl	10P	+1	17	[10z]
29	2	[Au_25_(PPh_3_)_10_(SePh)_5_Cl_2_](PF_6_)(SbF_6_)	Au_25_	*D*_5h_	*D*_5h_	0	179	5(μ‐Se)	2Cl	10P	+2	16	[10z]
30	2	[Au_25_(^*i*^Pr_2_‐bimy)_10_Br_7_](Cl)(NO_3_)	Au_25_	*D*_5(h)_	*D*_5(h)_	1	179	5(μ‐Br)	2Br	10C	+2	16	[10aa]
31	2	[Au_25_(^MesCH2^bimy)_10_Br_7_](Br)_2_	Au_25_	*D*_5_	*D*_5_	10	179	5(μ‐Br)	2Br	10C	+2	16	[10ab]
32	2	[Au_25_(^MesCH2^bimy)_10_Br_8_][B(C_6_F_5_)_4_]	Au_25_	*C*_2_	*D*_5_	17	179	2(Br) + 4(μ‐Br)	2Br	10C	+1	16	[10ab]
33	2	[Ag_31−*X*_Au_*X*_(dppm)_6_(S‐Adm)_6_Cl_7_]^2+^	Ag_17−*y*_Au_8+*y*_	*–*	*–*	–	–	2(μ‐S) + 4(μ‐Cl) + (μ^3^‐Cl)	2S	4S + 6P	+2	16	[10h]
34	2	[Au_29_Cd_2_(dppf)_2_(TBBT)_17_]^0^	Au_25_	*C*_2_	*D*_5h_	1	179	5(μ‐S)	2S	6S + 4P	0	16	[10ac]
35	2	[Ag_33_Pt_2_(dpt)_2_}_17_]^0^	Ag_23_Pt_2_	*C*_2_	*D*_5_	20	176	4S + 4(μ‐S)	2S	12S	0	16	[10ad]
36	3	[Ag_44_Pt_3_(dpt)_22_]^0^	Ag_34_Pt_3_	*C*_*i*_	*S*_10_	–	–	8S + 8(μ‐S)	2S	16S	0	22	[10ad]
37	3	[Au_37_(PPh_3_)_10_(PET)_10_X_2_]^+^	Au_37_	*D*_5d_	*D*_5d_	–	–	5(μ‐S)	2X	10P	+1	24	[10ae]
38	4	[Ag_61_(dpa)_27_](SbF_6_)_4_	Ag_49_	*C*_2_	*D*_5(d)_	–	–	30 N	2N	10N	+4	30	[10af]

a) SbF_6_ = hexafluoroantimonate ion, PF_6_ = hexafluorophosphate ion, Br = bromide ion, NO_3_ = nitrate ion, B(C_6_F_5_)_4_ = tetrakis(pentafluorophenyl)borate ion, X = halogen, Ni = nickel, Cd = cadmium.

b) The molecular symmetry of the framework structure.

c) Because the dihedral angle *φ* is close to 0° or 36° (=72°/2), some of these molecules exhibit imperfect symmetry: *h*, *d*, and *v* indicate a horizontal, dihedral, or vertical mirror plane, respectively. The symmetry was not determined in the case that the synthesis gave a mixture of products.

d) The torsion angle (the average dihedral angle) between the two M_13_ groups (see Figure 3).

e) The bond angle between the two M_13_ groups (see Figure 3).

f) The charge.

g) The number of valence electrons.

h) A summed point group indicates that the product was a mixture of molecules for which the symmetries were *C*_5v_ and *C*_s_.

### 2.1 M_25_ Protected by PR_3_ and X Ligands

**Figure 5:**
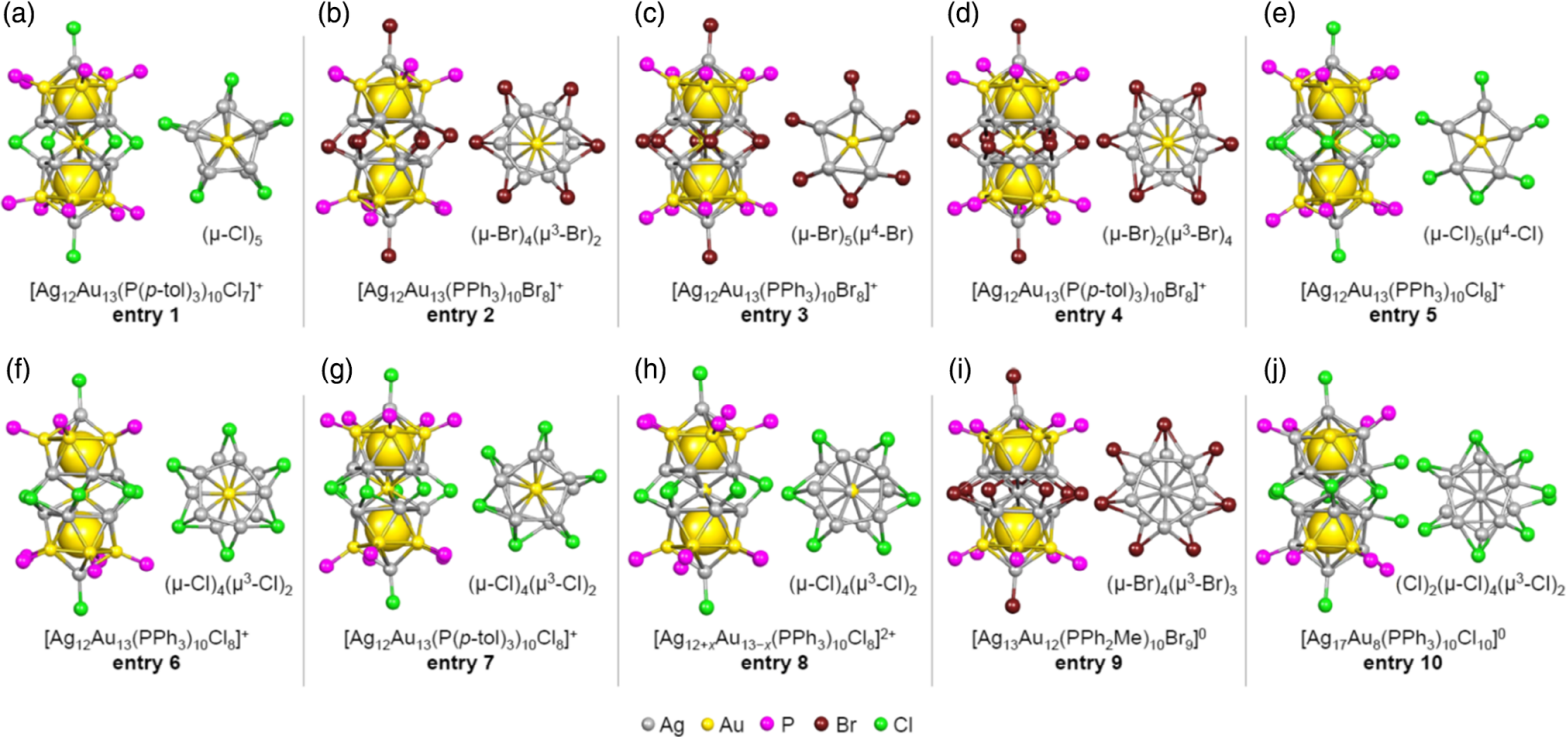
Framework structures of bimetallic M_25_ superatomic molecules protected by PR_3_ and X ligands. a) [Ag_12_Au_13_(P(*p*‐tol)_3_)_10_Cl_7_]^+^ (entry 1),^[10a]^ b) [Ag_12_Au_13_(PPh_3_)_10_Br_8_]^+^ (entry 2),^[10b]^ c) [Ag_12_Au_13_(PPh_3_)_10_Br_8_]^+^ (entry 3),^[10c]^ d) [Ag_12_Au_13_(P(*p*‐tol)_3_)_10_Br_8_]^+^ (entry 4),^[10d]^ e) [Ag_12_Au_13_(PPh_3_)_10_Cl_8_]^+^ (entry 5),^[10e,f]^ f) [Ag_12_Au_13_(PPh_3_)_10_Cl_8_]^+^ (entry 6),^[10f]^ g) [Ag_12_Au_13_(P(*p*‐tol)_3_)_10_Cl_8_]^+^ (entry 7),^[10g]^ h) [Ag_12+*x*_Au_13−*x*_(PPh_3_)_10_Cl_8_]^2+^ (entry 8),^[10h]^ i) [Ag_13_Au_12_(PPh_2_Me)_10_Br_9_]^0^ (entry 9),^[10i]^ and j) [Ag_17_Au_8_(PPh_3_)_10_Cl_10_]^0^ (entry 10).^[10j]^ In each structure, the left and right images indicate a side and top view, respectively. In the top views, only the M_C1_ and M_L1_ site metal atoms and L_1_ atoms are shown for the clarity. These structures are classified by the number of halogen ligands and bonding motifs.

Since the 1980s, Teo et al. have successfully synthesized a number of novel M_25_ superatomic molecules and determined the corresponding geometric structures using single‐crystal X‐ray diffraction (SC‐XRD). In many cases, these structures incorporated PR_3_ and X ligands, and the superatomic molecules were obtained by reducing metal salts (that is, complexes consisting of metal ions, PR_3_ and/or X) or smaller metal clusters. **Figure** **5**a–e,g,i provides the geometric structures of representative M_25_ superatomic molecules fabricated in this prior work (**entries 1–5**, **7,** and **9**). Interestingly, the number of L_1_ ligands and the bridging structures in these molecules were found to vary with changes in the type of ligand and the counter ion. These phenomena were attributed to the steric effects of the various ligands as well as to electronic factors.^[^ ^9^ ^]^ The M_25_ core was also found to undergo torsion depending on the type and number of L_1_ and L_2_ ligands, producing various symmetries in the core, such as *D*_5h_ or *D*_5d_ symmetries or their subgroups (Table 1). Additionally, the charge state of the metal cluster changed from +2 to 0 as the number of L_1_ ligands was increased. In the case of an M_25_ superatomic molecule, a closed‐shell electronic structure (2 × (1S^2^1P^6^)) was obtained when the total number of valence electrons was 16.^[^ ^17^ ^]^ Therefore, varying the quantity of L_1_ ligands (anionic ligands) produced different charge states in the metal clusters. For **entry 6** (Figure 5f), a selective synthesis method was established in a later study by Jin et al.^[10f]^ Note that this structure is isomeric with **entry 5**. Jin demonstrated a reversible structural transition between these isomers based on temperature, suggesting that M_25_ superatomic molecules could have applications as molecular motors.^[10f]^ Zhu et al. reported the synthesis of [Ag_12+*x*_Au_13−*x*_(PPh_3_)_10_Cl_8_]^2+^ (**entry 8**) (Figure 5h), in which the M_C1_ site is either Au or Ag.^[10h]^ The L_1_ coordination environment in this superatomic molecule is similar to that in [Ag_12_Au_13_(PPh_3_)_10_Br_8_]^+^ (**entry 2**). Liu et al. produced the superatomic molecule [Ag_17_Au_8_(PPh_3_)_10_Cl_10_]^0^ (**entry 10**) (Figure 5j), which had a different geometric structure compared with those in **entries 1–9**.^[10j]^ In this structure, eight Ag atoms in the Ag_25_ cluster were replaced with Au atoms such that the M_25_ core did not have fivefold symmetry. In addition, two Cl atoms were terminally coordinated to the metal atom at the M_L1_ site without bridging, in contrast to the metal clusters in **entries 1–9**. The bond angle (*θ*) between the M_13_ units in this structure was 173°, meaning that the long axis exhibits a greater degree of bending than those in the other superatomic molecules.^[10j]^

**Figure 6:**
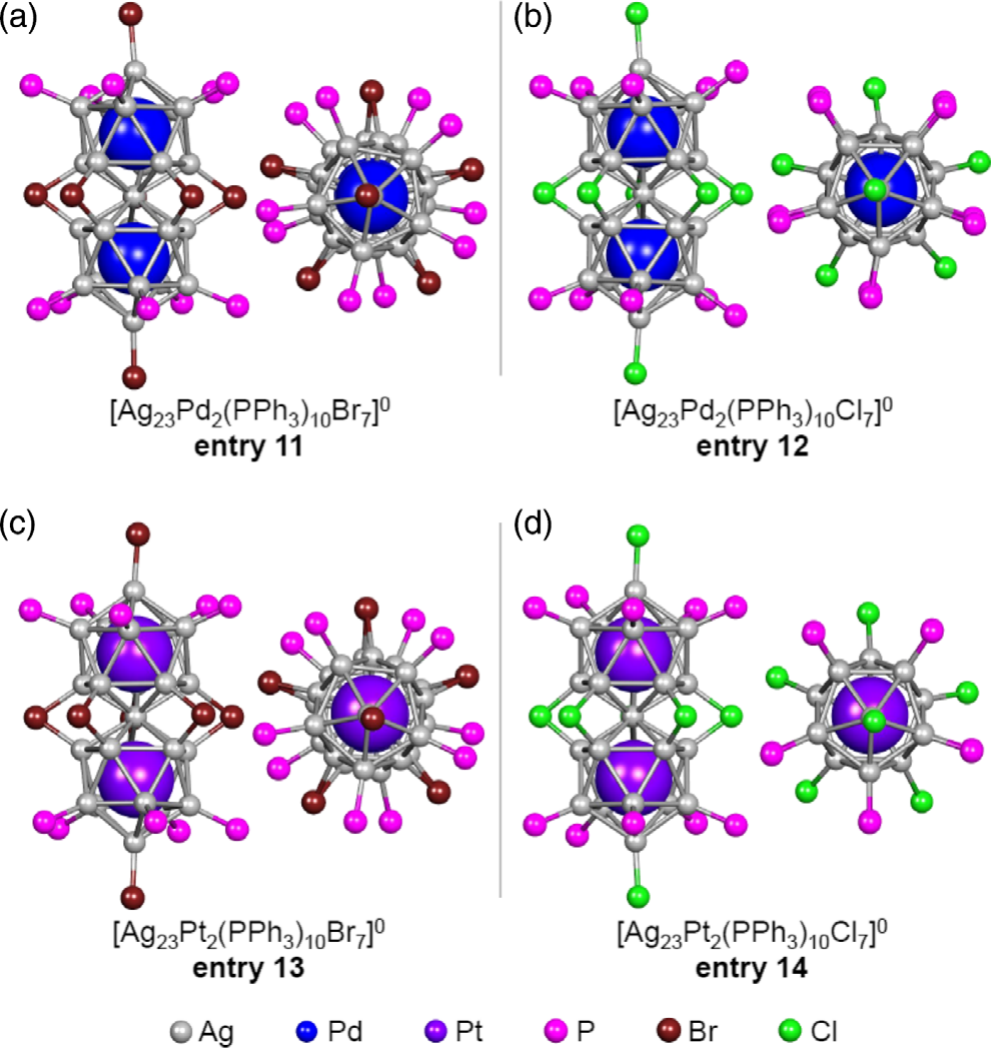
Framework structures of Ag_23_M_2_ cores protected by PPh_3_ and X ligands. a) [Ag_23_Pd_2_(PPh_3_)_10_Br_7_]^0^ (**entry 11**),^[10k]^ b) [Ag_23_Pd_2_(PPh_3_)_10_Cl_7_]^0^ (**entry 12**),^[10l]^ c) [Ag_23_Pt_2_(PPh_3_)_10_Br_7_]^0^ (**entry 13**),^[10k]^ and d) [Ag_23_Pt_2_(PPh_3_)_10_Cl_7_]^0^ (**entry 14**).^[10m]^ In each structure, the left and right figures indicate the side and top view, respectively.

Bakr et al. produced the Ag‐based superatomic molecule [Ag_23_Pt_2_(PPh_3_)_10_Cl_7_]^0^ (**entry 14**)^[10m]^ in which the two central Ag atoms of each Ag_13_ cluster were replaced with Pt atoms. The author's group also recently synthesized three novel Ag‐based superatomic molecules having the formula [Ag_23_M_2_(PPh_3_)_10_X_7_]^0^ (M/X = Pd/Br (**entry 11**); Pd/Cl (**entry 12**); Pt/Br (**entry 13**)) (**Figure** **6**).^[10k,l]^ In the case of **entry 13** ([Ag_23_Pt_2_(PPh_3_)_10_Br_7_]^0^), as in **entry 14** ([Ag_23_Pt_2_(PPh_3_)_10_Cl_2_]^0^), the two central Ag atoms of the Ag_13_ cluster were replaced with Pt atoms although the L_1_ and L_2_ ligands were Br rather than Cl (Figure 6c,d). **Entry 14** had an achiral geometric structure because its M_25_ core exhibited a mirror plane. In contrast, in **entry 13** the dihedral angle *φ* between the two Ag_12_Pt cores was twisted by approximately 10° such that there was no mirror plane in the M_25_ core or in the entire molecule, leading to chirality. Although the Ag−Br bond is longer than the Ag−Cl bond, the distance between the two Ag_12_Pt units in this structure was kept by this torsion. Similar torsion was also observed in **entry 11** ([Ag_23_Pd_2_(PPh_3_)_10_Br_7_]^0^). Studies have shown that the stability of these Ag_23_M_2_ superatomic molecules having Ag as the base element together with PR_3_ and X as ligands (**entries 11–14**) can be greatly improved by using Cl atoms (which have a high affinity for Ag) as the L_1_ ligands and by replacing Ag atoms with other metals to strengthen the metallic bonds and thereby form a more rigid metallic core.^[10k,l]^

**Figure 7:**
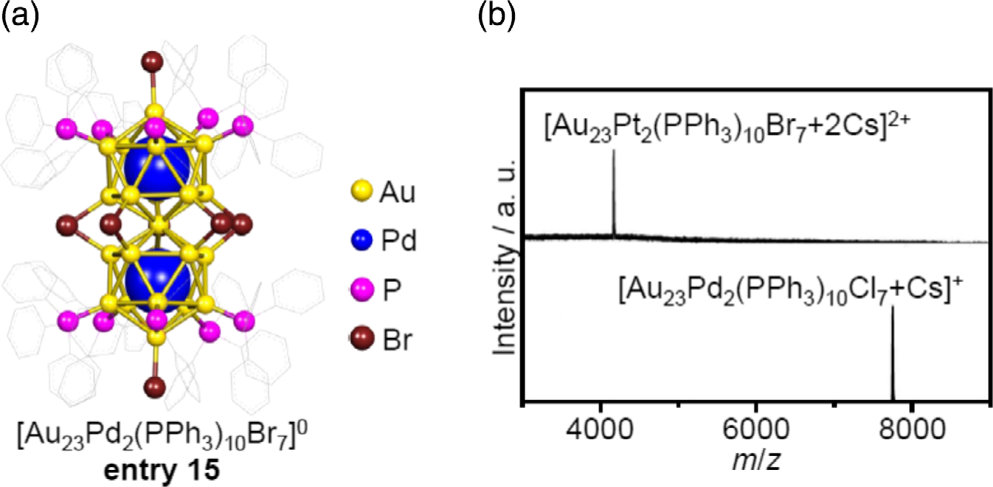
a) The geometrical structure of [Au_23_Pd_2_(PPh_3_)_10_Br_7_]^0^ (**entry 15**).^[10n]^ b) Electrospray ionization‐mass spectra of [Au_23_Pt_2_(PPh_3_)_10_Br_7_]^0^ and [Au_23_Pd_2_(PPh_3_)_10_Cl_7_]^0^. b) Reproduced with permission.^[10n]^ Copyright 2017, American Chemical Society.

Zhu et al. reported the synthesis of [Au_23_Pd_2_(PPh_3_)_10_Br_7_]^0^ (**entry 15**) with Au as the base element instead of Ag (**Figure** **7**a). Both Cl‐substituted ([Au_23_Pd_2_(PPh_3_)_10_Cl_7_]^0^) and Pt‐substituted ([Au_23_Pt_**2**_(PPh_3_)_10_Br_7_]^0^) analogs were also obtained (Figure 7b), although the geometric structures of these compounds were not been determined by SC‐XRD.^[10n]^ To the best of our knowledge, there have been no reports of [Au_25_(PR_3_)_10_X_7_]^+^ molecules with cores composed solely of Au. It is thought that an X‐bridged Au_25_ superatomic molecule would not be sufficiently stable to be isolated unless the metal core was strengthened by doping with different metals.

**Figure 8:**
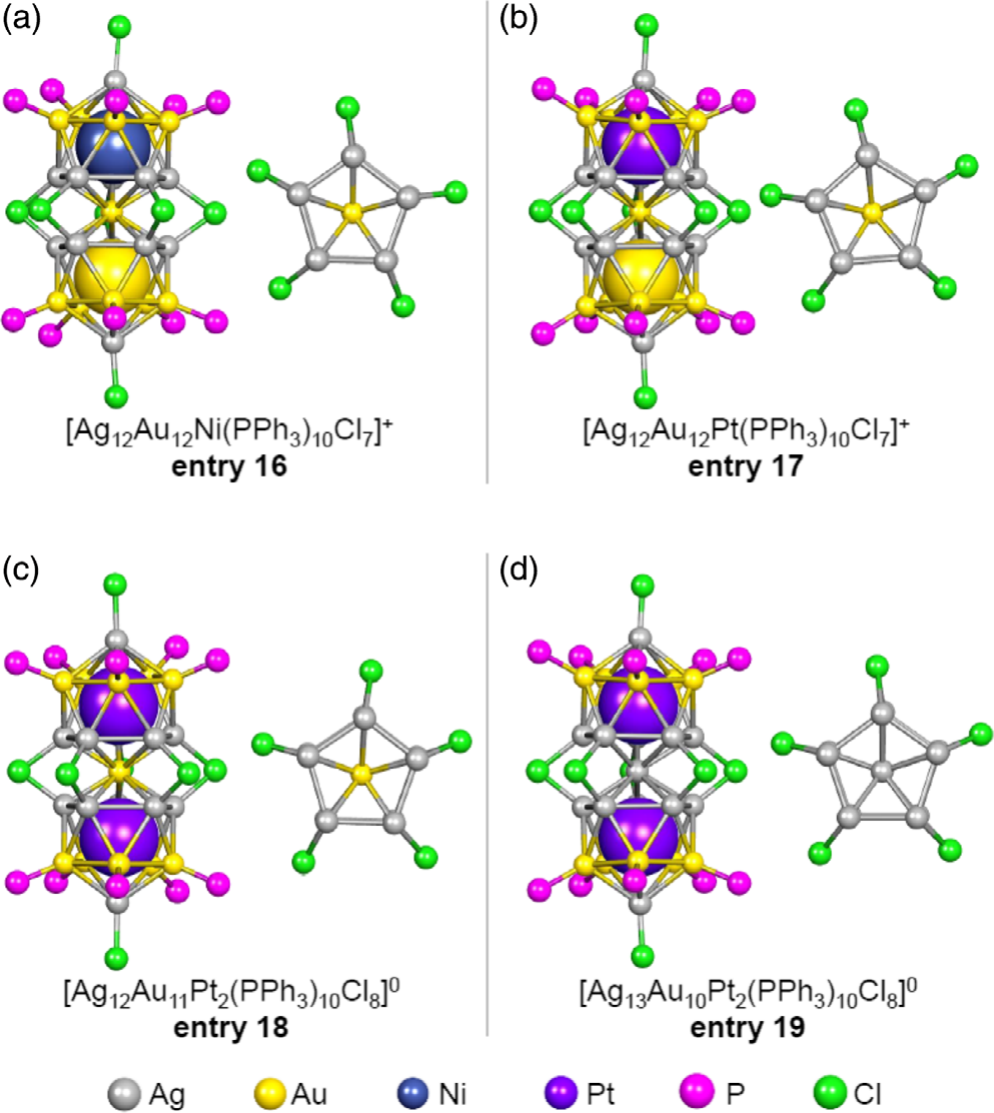
Framework structures of trimetallic M_25_ superatomic molecules. a) [Ag_12_Au_12_Ni(PPh_3_)_10_Cl_7_]^+^ (entry 16),^[10o]^ b) [Ag_12_Au_12_Pt(PPh_3_)_10_Cl_7_]^+^ (entry 17),^[10p]^ c) [Ag_12_Au_11_Pt_2_(PPh_3_)_10_Cl_7_]^0^ (entry 18),^[10q]^ and d) [Ag_13_Au_10_Pt_2_(PPh_3_)_10_Cl_7_]^0^ (entry 20).^[10r]^

In addition to the M_25_ superatomic molecules containing two metal elements described above, Teo et al. and Steggerda et al. have also reported the synthesis of several structures incorporating three metals (**entries 16–19**; **Figure** **8**).^[10o-r]^ These studies established the existence of preferential sites for each metal element.^[^ ^9^ ^]^

### 2.2 M_25_ Protected by PR_3_, ER, and X Ligands

**Figure 9:**
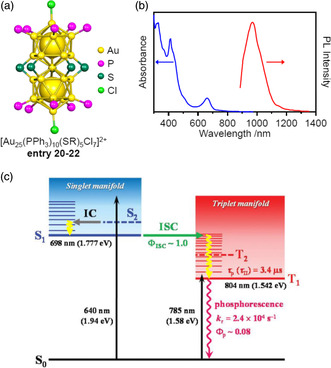
a) Framework structure of [Au_25_(PPh_3_)_10_(SR)_5_Cl_2_]^2+^ (SR = SEt (entry 20),^[10s]^ PET (entry 21),^[10t]^ or SPh (entry 22)^[10u]^). b) UV–visible absorption/PL spectra of [Au_25_(PPh_3_)_10_(PET)_5_Cl^2^]^2+^. b) Reproduced with permission.^[^ ^23^ ^]^ Copyright 2021, Wiley‐VCH. c) Excited state deactivation pathway of [Au_25_(PPh_3_)_10_(SR)_5_Cl_2_]^2+^. Reproduced with permission.^[^ ^24^ ^]^ Copyright 2022, Royal Society of Chemistry.

Au and Ag can form strong bonds with thiolate (SR) ligands.^[^ ^20^ ^]^ Therefore, in the case that such ligands are included in superatomic molecules, the structure tends to be relatively stable. Tsukuda et al. produced [Au_25_(PPh_3_)_10_(SR)_5_Cl_2_]^2+^ (R = C_*n*_H_2*n*+1_; *n* = 2 for **entry 20**) containing only Au in the metal core using SR ligands in addition to PR_3_ and X (**Figure** **9**a).^[10s]^ In this structure, the SR ligands were coordinated to the metal atoms at the M_L1_ sites in the form of bridges (μ‐SR) while the Cl atoms were coordinated at the M_L2_ sites. Subsequently, the syntheses of [Au_25_(PPh_3_)_10_(SR)_5_Cl_2_]^2+^ species, in which different functional groups were contained in the SR ligands, were reported by Jin et al., Li et al., and Klinke et al. These ligands comprised 2‐phenylethanethiolate (PET) (see also Figure 4b) in the case of **entry 21** (Figure 9)[^10^, ^21^] and benzenethiolate (SPh) (Figure 4c) in the case of **entry 22.** ^[10u]^

These [Au_25_(PPh_3_)_10_(SR)_5_Cl_2_]^2+^ molecules were also studied extensively with respect to their optical properties and electronic structures.^[^ ^22^ ^]^ As a result, [Au_25_(PPh_3_)_10_(SR)_5_Cl_2_]^2+^ was found to absorb light over a wide range from the near‐infrared region (approximately 900 nm) to the ultraviolet (UV) region^[10s]^ and also to exhibit photoluminescence (PL) at approximately 990 nm^[^ ^23^ ^]^ (Figure 9b). Analyses of the PL characteristics of [Au_25_(PPh_3_)_10_(PET)_5_Cl_2_]^2+^ (Figure 9c) found a quantum yield (Φ_PL_) and lifetime (*τ*_PL_) of approximately 8% and 3.2 μs, respectively, indicating that this molecule could be better suited to certain applications compared with other secondary near‐infrared chromophores.^[^ ^23^ ^]^ The spin multiplicity of the excited state of [Au_25_(PPh_3_)_10_(PET)_5_Cl_2_]^2+^ was recently reported to be 3 by Mitsui et al.^[^ ^24^ ^]^ The same group also showed that the intersystem crossing yield (Φ_ISC_) in [Au_25_(PPh_3_)_10_(PET)_5_Cl_2_]^2+^ is approximately 1 using the triplet‐triplet annihilation‐based photon upconversion phenomenon in conjunction with fluorescent dyes. These results suggest that [Au_25_(PPh_3_)_10_(SR)_5_Cl_2_]^2+^ could be utilized as a phosphorescent material with a dark‐excited singlet state and a bright‐excited triplet state at room temperature.^[^ ^24^ ^]^ The [Au_25_(PPh_3_)_10_(SR)_5_Cl_2_]^2+^ species have also been reported to exhibit various catalytic activities, including electrochemical CO_2_ reduction catalysis, selective aerobic oxidation photocatalysis of amines to imines, and aerobic oxidation of glucose to gluconic acid.[^10^, ^25^]

**Figure 10:**
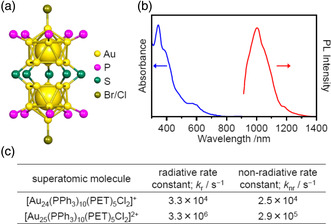
a) Framework structure of [Au_24_(PPh_3_)_10_(PET)_5_X_2_]^+^ (X = Br or Cl)^[^ ^26^ ^]^ and b) UV–vis absorption/PL spectra of [Au_24_(PPh_3_)_10_(PET)_5_Cl_2_]^+^. b) Reproduced with permission.^[^ ^23^ ^]^ Copyright 2021, Wiley‐VCH. c) The effect of a central Au atom on the excitation relaxation rate.^[^ ^23^ ^]^

The syntheses of [Au_24_(PPh_3_)_10_(SR)_5_X_2_]^+^ (X = Br or Cl) molecules lacking an Au atom at the M_C1_ site were reported by Jin et al. (**Figure** **10**a).^[^ ^26^ ^]^ Both [Au_25_(PPh_3_)_10_(PET)_5_Cl_2_]^2+^ and [Au_24_(PPh_3_)_10_(PET)_5_X_2_]^+^ had similar geometric structures (Figure 9a and 10a) together with very similar light absorption and PL characteristics (Figure 9b and 10b).^[^ ^23^, ^27^ ^]^ However, the Φ_PL_ of [Au_24_(PPh_3_)_10_(PET)_5_X_2_]^+^ was only on the order of 1% and so was extremely low compared with that of [Au_25_(PPh_3_)_10_(PET)_5_Cl_2_]^2+^. Li et al. studied the excited states of these two metal clusters in detail and concluded that the lack of a central Au in [Au_25_(PPh_3_)_10_(PET)_5_Cl_2_]^2+^ reduced the nonradiative rate constant to approximately 11%, resulting in the low Φ_PL_ of [Au_24_(PPh_3_)_10_(PET)_5_X_2_]^+^ compared with that of [Au_25_(PPh_3_)_10_(PET)_5_Cl_2_]^2+^ (Figure 10c).^[^ ^23^ ^]^

**Figure 11:**
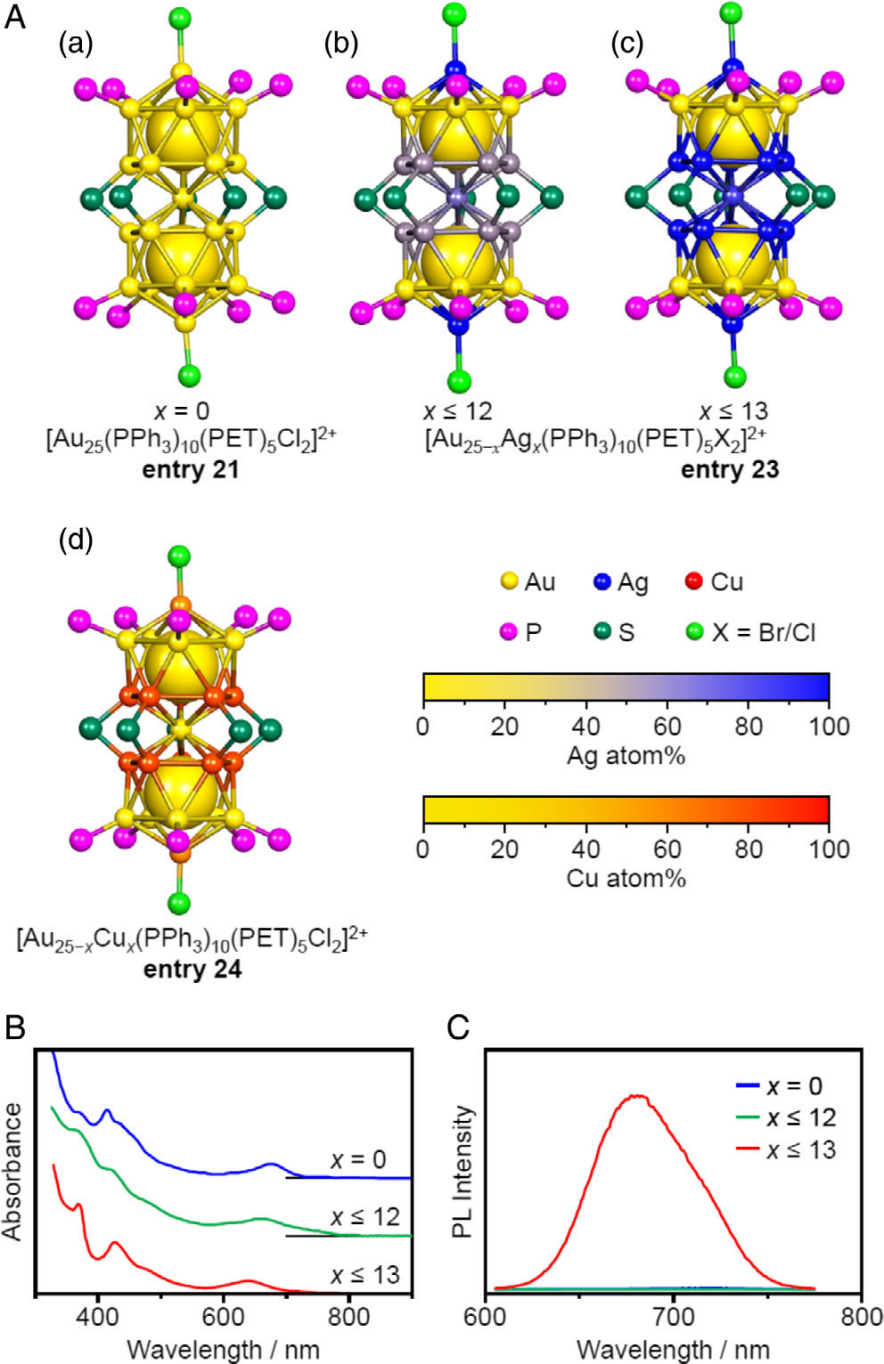
A) Framework structures of: a) [Au_25_(PPh_3_)_10_(PET)_5_Cl_2_]^2+^ (entry 21),^[10t]^ b), [Ag_*x*_Au_25−*x*_(PPh_3_)_10_(PET)_5_X_2_]^2+^ (*x* ≤ 12), c) [Ag_*x*_Au_25−*x*_(PPh_3_)_10_(PET)_5_X_2_]^2+^ (*x* ≤ 13; entry 23),^[10v]^ and d) [Au_25−*x*_Cu_*x*_(PPh_3_)_10_(PET)_5_Cl_2_]^2+^ (entry 24).^[10w]^ B) UV–vis absorption and C) PL spectra of [Ag_*x*_Au_25−*x*_(PPh_3_)_10_(SR)_5_X_2_]^2+^. Data in (B,C) are replotted from ref. [10v].

The alloying strategy has also been implemented for [Au_25_(PPh_3_)_10_(SR)_5_Cl_2_]^2+^. Jin et al. and Zhu et al. synthesized [Ag_*x*_Au_25−*x*_(PPh_3_)_10_(PET)_5_Cl_2_]^2+^ (*x* ≤ 13, **entry 23**) and [Au_25−*x*_Cu_*x*_ (PPh_3_)_10_(PET)_5_Cl_2_]^2+^ (**entry 24**), in which multiple Au atoms were replaced with Ag or Cu atoms, and investigated the Ag and Cu substitution sites (**Figure** **11**A).^[10v,w]^ The results showed that in the case of [Ag_*x*_Au_25−*x*_(PPh_3_)_10_(PET)_5_Cl_2_]^2+^, Ag atoms were substituted at the M_L1_ or M_L2_ sites up to the 12^th^ Ag, while the 13^th^ Ag was substituted at an M_C1_ site.^[10v]^ In [Au_25−*x*_Cu_*x*_(PPh_3_)_10_(PET)_5_Cl_2_]^2+^, Cu was substituted at the M_L1_ and M_L2_ sites.^[10w]^ The absorption spectra of [Ag_*x*_Au_25−*x*_(PPh_3_)_10_(PET)_5_Cl_2_]^2+^ gradually changed when the number of Ag atoms increased (Figure 11B). In the same work, they found that the substitution of up to 12 Ag atoms did not significantly change the PL properties of [Ag_*x*_Au_25−*x*_(PPh_3_)_10_(PET)_5_Cl_2_]^2+^, while the Φ_PL_ of the molecule was increased to approximately 40% after the 13th Ag was substituted at the M_C1_ site (Figure 11C). Using density functional theory (DFT) calculations, Muniz‐Miranda et al. reported that the oscillator strength of the *S*_0_ → *S*_1_ transition was enhanced when the 12th or 13th Au atom in [Au_25_(PPh_3_)_10_(PET)_5_Cl_2_]^2+^ was replaced.^[^ ^28^ ^]^ Mitsui et al. reported that these emission characteristics could be attributed to phosphorescence and that the 13th Ag substitution shifted the excited triplet state to a higher energy, thereby suppressing the *T*_1_ → *S*_0_ intersystem crossing, such that **entry 23** exhibited a higher Φ_PL_ value than [Ag_*x*_Au_25−*x*_(PPh_3_)_10_(PET)_5_Cl_2_]^2+^ (*x* ≤ 12).^[^ ^29^ ^]^

**Figure 12:**
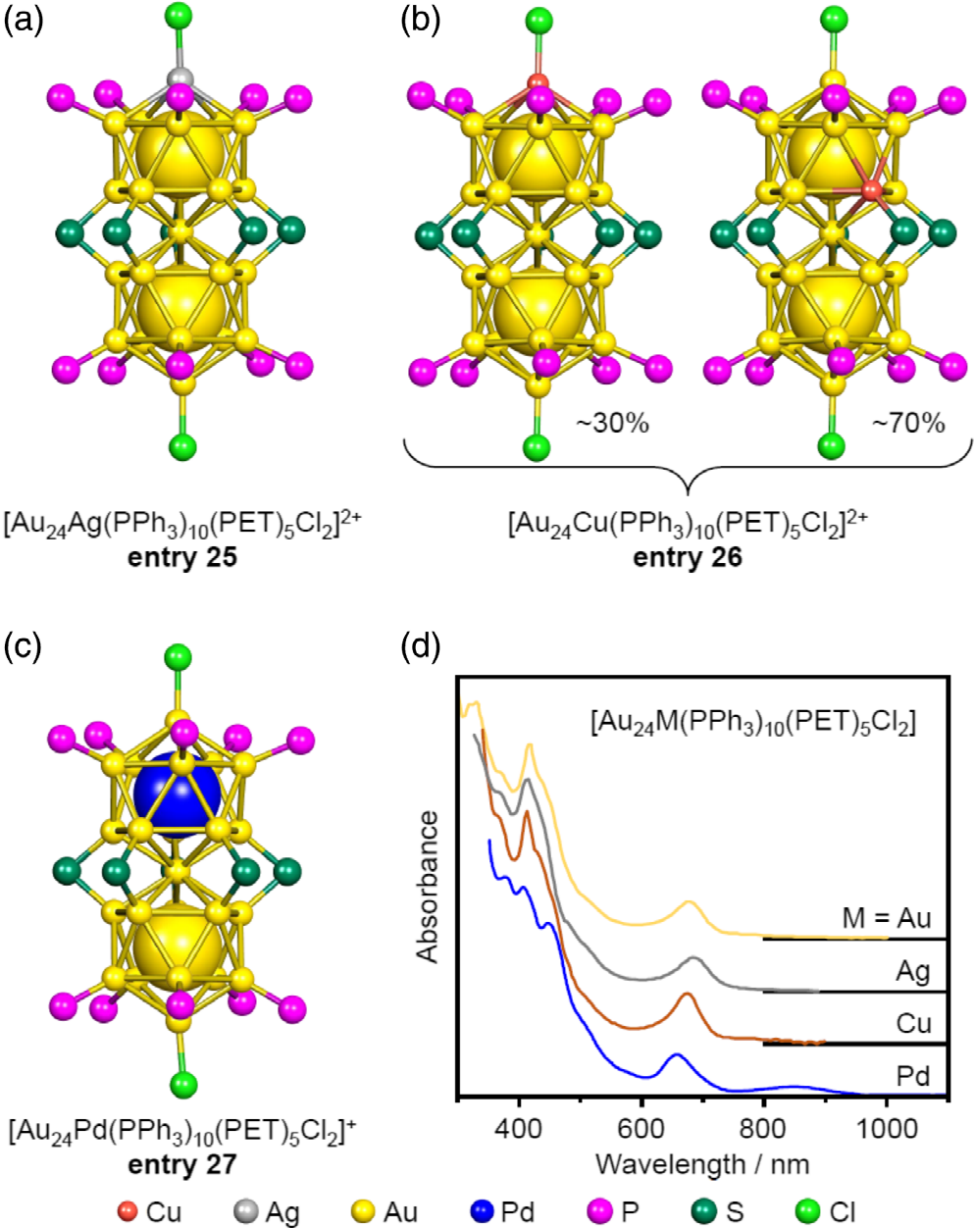
Framework structures of monometal doped [Au_24_M(PPh_3_)_10_(PET)_5_Cl_2_]^*z*^. a) [AgAu_24_(PPh_3_)_10_(PET)_5_Cl_2_]^2+^ (entry 25),^[10x]^ b) [Au_24_Cu(PPh_3_)_10_(PET)_5_Cl_2_]^2+^ (entry 26),^[10x]^ and c) [Au_24_Pd(PPh_3_)_10_(PET)_5_Cl_2_]^+^ (entry 27).^[10y]^ d) Comparison of UV–vis absorption spectra of [Au_24_M(PPh_3_)_10_(PET)_5_Cl_2_]^*z*^ incorporating various metals. Data in (d) are replotted from refs. [10x] (yellow, gray, and brown lines) and ref. [10y] (blue line).

During the synthesis of the [Au_25−*x*_M_*x*_(PPh_3_)_10_(PET)_5_Cl_2_]^2+^ (M = Ag, Cu) molecules noted above, the number of Ag or Cu atoms that were inserted could not be controlled with atomic precision. However, in later work, Jin et al. synthesized [Au_24_M(PPh_3_)_10_(PET)_5_Cl_2_]^2+^ (M = Ag (**entry 25**), Cu (**entry 26**)) in which one Au atom in [Au_25_(PPh_3_)_10_(PET)_5_Cl_2_]^2+^ was replaced with an Ag or Cu atom (**Figure** **12**a,b).^[10x]^ SC‐XRD analyses revealed that Ag insertion occurred at the M_L2_ site of the Au_25_ group while Cu insertion took place at the M_L1_ or M_L2_ sites. Consequently, the Cu‐substituted form was obtained as a mixture of two isomers (Figure 12b). The same group also demonstrated the precise synthesis of [Au_23_Ag_2_(PPh_3_)_10_(PET)_5_Cl_2_]^2+^, whose Ag atoms are doped into the M_L2_ sites by controlling the reaction mechanism.^[10x]^ We also synthesized [Au_24_Pd(PPh_3_)_10_(PET)_5_Cl_2_]^+^ (**entry 27**), in which only one Au atom at the M_C2_ site of [Au_25_(PPh_3_)_10_(PET)_5_Cl_2_]^2+^ was replaced with a Pd atom (Figure 12c).^[10y]^ Because Pd differs from Au by one group in the periodic table, **entry 27** was synthesized in the charge state of +1, unlike [Au_25_(PPh_3_)_10_(PET)_5_Cl_2_]^2+^ (**entry 21**). In the case of the former, unlike the latter, the absorption peak at longer wavelengths was clearly split into two peaks (Figure 12d). This splitting was shown by DFT calculations performed by Jiang et al. to be attributed to the splitting of the orbital near the highest occupied molecular orbital (HOMO) due to the monoatomic Pd doping.^[10y]^ **Entry 27** has also been found to induce a permanent dipole moment in superatomic molecule, and so could possibly be used as a molecular rectifier or dipole material.^[10y]^

**Figure 13:**
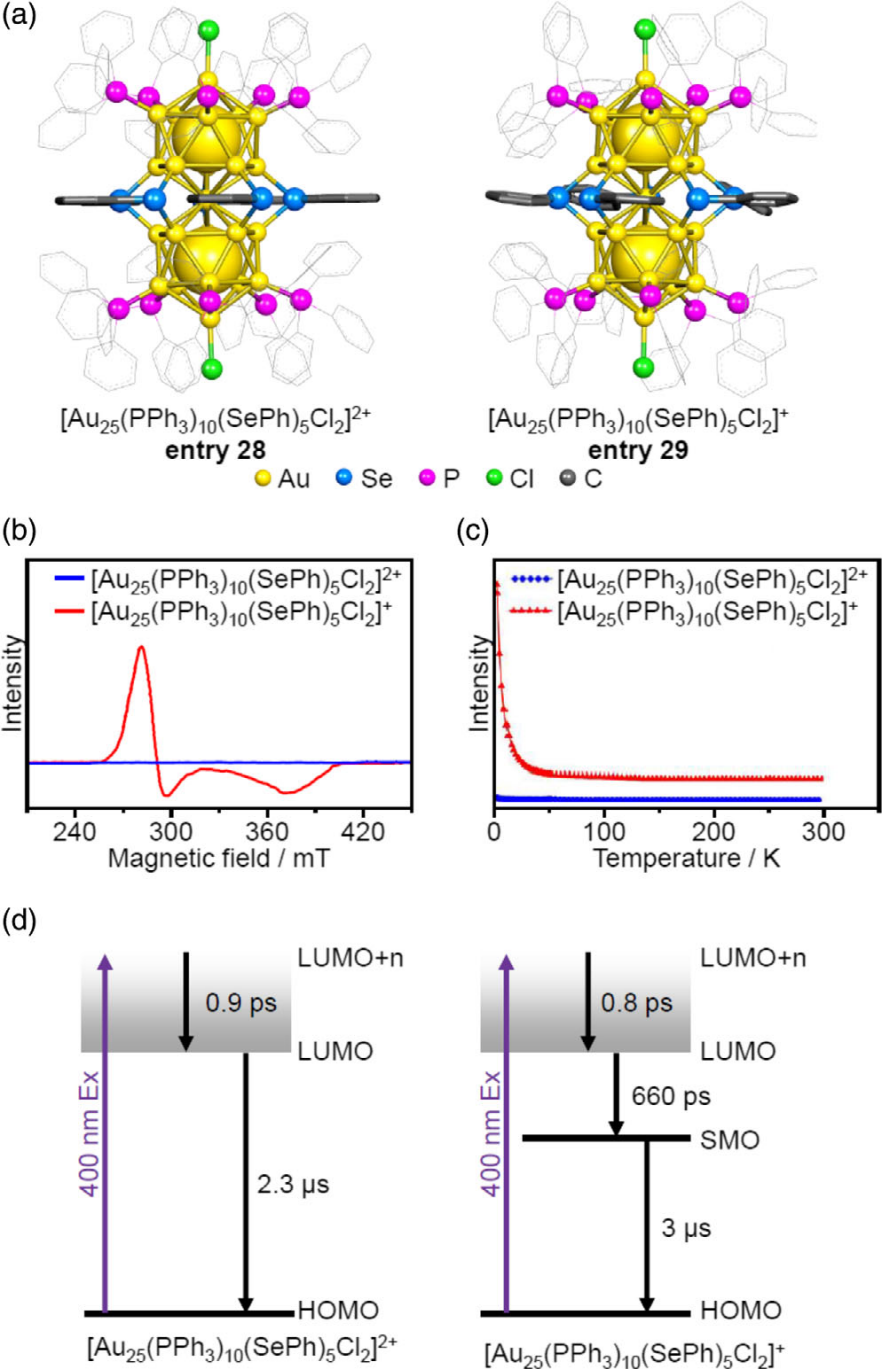
a) Geometrical structures, b) EPR spectra at 5 K, and c) magnetic susceptibilities versus temperature plots for [Au_25_(PPh_3_)_10_(SePh)_5_Cl_2_]^2+/+^ (entries 28 and 29).^[10z]^ b,c) Reproduced with permission.^[10z]^ Copyright 2016, American Chemical Society. d) Excited state relaxation dynamics for these same molecules. Reproduced with permission.^[^ ^30^ ^]^ Copyright 2021, The Royal Society of Chemistry.

Zhu et al. synthesized [Au_25_(PPh_3_)_10_(SePh)_5_Cl_2_]^2+/+^ (SePh = benzeneselenolate (Figure 4d); **entries 28** and **29**) molecules, each of which had a selenolate (SeR) ligand at the L_1_ site (**Figure** **13**a).^[10z]^ Both these materials were synthesized using PPh_3_‐protected Au clusters (Au_*n*_(PPh_3_)_*m*_) as a precursor while controlling the charge state of the product by optimizing the reaction temperature, the amount of reducing agent, the solvent, and the amount of benzeneselenol (PhSeH). The geometrical structures of these molecules were similar to one another, although the arrangements of the Ph groups attached to the Se atoms differed (Figure 13a). In addition, the structure in **entry 28** had a closed‐shell electronic structure while that in **entry 29** contained one extra electron. Therefore, the electron paramagnetic resonance spectrum of the latter had peaks related to *S* = 1/2 at *g* values of 2.40, 2.26, and 1.78 (Figure 13b,c), and the magnetic properties of Au_25_(PPh_3_)_10_(SePh)_5_Cl_2_ could be tuned by varying the charge state. Zhou et al. investigated the relaxation dynamics of excited states in closed and open shell electron systems by acquiring transient absorption spectra and by investigating the transient absorption anisotropy of the [Au_25_(PPh_3_)_10_(SePh)_5_Cl_2_]^2+/+^ molecules. The results demonstrated that the relaxation lifetimes of less than 1 ps and ≈1 μs were associated with the internal conversion from higher to lower excited state and lower excited state to ground state, respectively. However, an intermediate time constant of approximately ≈100 ps was observed only in the case of the open‐shell system and originated from the presence of singly occupied molecular orbital (SMO) (Figure 13d).^[^ ^30^ ^]^ DFT calculations have also suggested that ligand exchange reactions may occur between these M_25_ superatomic molecules in solution.^[^ ^31^ ^]^

### 2.3 M_25_ Protected by PR_3_, C ≡ CR, and X Ligands

**Figure 14:**
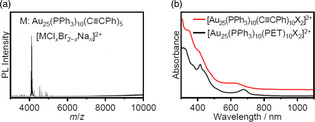
a) The electrospray ionization mass spectrum of [Au_25_(PPh_3_)_10_(PA)_5_X_2_]^2+^ and b) the UV–vis absorption spectra of [Au_25_(PPh_3_)_10_(PET)_5_X_2_]^2+^ and [Au_25_(PPh_3_)_10_(PA)_5_X_2_]^2+^. a,b) Reproduced with permission.^[14r]^ Copyright 2014, American Chemical Society.

The synthesis of M_25_ superatomic molecules containing acetylide ligands, C ≡ CR, has thus far been limited to [Au_25_(PPh_3_)_10_(PA)_5_X_2_]^2+^ (PA = phenylacetylide), which was reported by Jin et al. in 2014.^[14r]^ The formation of this molecule was confirmed by electrospray ionization‐mass spectrometry and optical absorption spectroscopy (**Figure** **14**). A geometrical structure in which the SR ligands at the L_1_ sites in [Au_25_(PPh_3_)_10_(SR)_5_X_2_]^2+^ were replaced by PA ligands was predicted.^[14r]^ Thus, the C ≡ CR ligand evidently appears in area A in the ligand classification system, as shown in Figure 4. The same group also found that [Au_25_(PPh_3_)_10_(PA)_5_X_2_]^2+^ could be utilized as a highly efficient and selective catalyst for the conversion of terminal alkynes to alkenes.^[14r]^

### 2.4 M_25_ Protected by NHC and X Ligands

**Figure 15:**
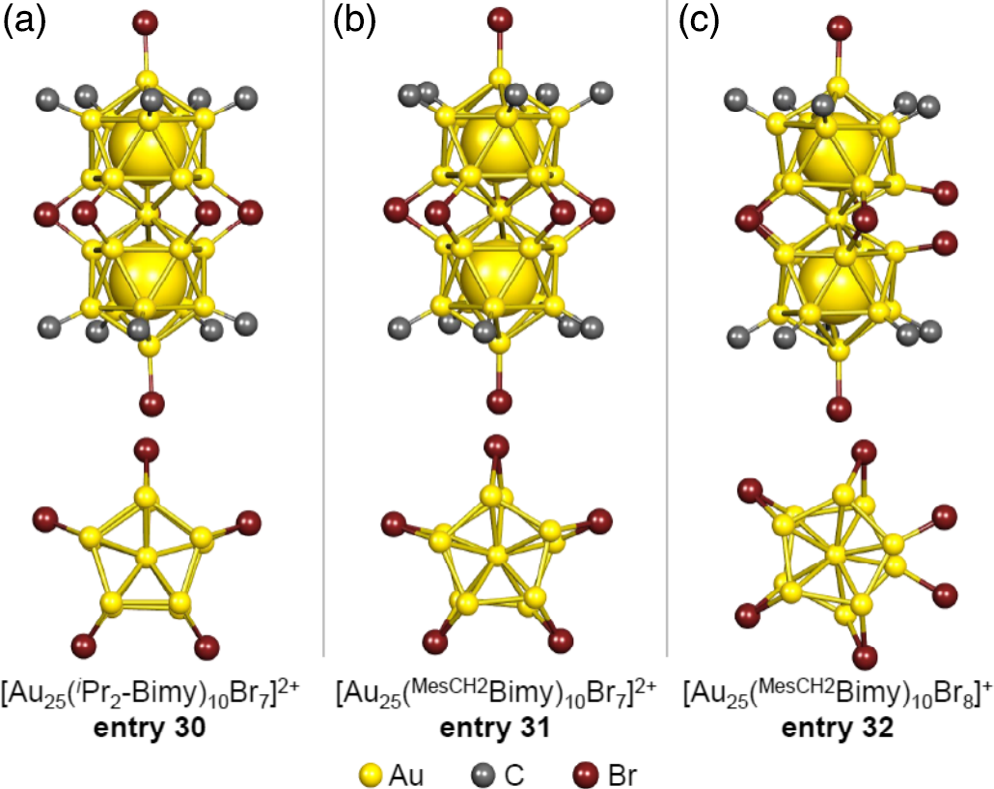
Framework structures of NHC‐protected Au_25_ superatomic molecules. a) [Au_25_(^*i*^Pr_2_‐bimy)_10_Br_7_]^2+^ (entry 30),^[10aa]^ b) [Au_25_(^MesCH2^bimy)_10_Br_7_]^2+^ (entry 31), and c) [Au_25_(^MesCH2^bimy)_10_Br_8_]^+^ (entry 32).^[10ab]^ Side views of the main structures and top views around the M_C1_, M_L1_, and L_1_ atoms are depicted in the upper and lower part of each figure, respectively.

PR_3_ moieties have been used as L_3_ ligands in M_25_ superatomic molecules, as described in Section 2.1, 2.2, 2.3. However, several M_25_ superatomic molecules having NHC as the L_3_ ligand have also been reported in recent years. Zheng et al. synthesized [Au_25_(^*i*^Pr_2_‐bimy)_10_Br_7_]^2+^ (^*i*^Pr_2_‐bimy = 1,3‐di**‐** *i* **‐**propylbenzimidazol‐2‐ylidene (Figure 4j); **entry 30**) by reducing Au‐NHC complexes and Au‐SMe_2_Cl complexes (SMe_2_ = dimethyl sulfide) using sodium borohydride (**Figure** **15**a).^[10aa]^ In the resulting [Au_25_(^*i*^Pr_2_‐bimy)_10_Br_7_]^2+^, the NHC carbon was coordinated to the metal atom at the M_L3_ site. Interestingly, superatomic molecules containing NHC ligands ([Au_25_(^*i*^Pr_2_‐bimy)_10_Br_7_]^2+^) exhibited higher stability than those containing SR and PR_3_ ligands ([Au_25_(PPh_3_)_10_(PET)_5_Cl_2_]^2+^; **entry 21**). Because the Au−C bond is stronger than the Au−P bond,^[^ ^32^ ^]^ those molecules containing the former bonds could be more robust.^[10aa]^ The [Au_25_(^*i*^Pr_2_‐bimy)_10_Br_7_]^2+^ molecule has also been shown to function as a highly active catalyst for the cyclic isomerization of alkynyl amines to indoles.^[10aa]^

The [Au_25_(^MesCH2^bimy)_10_Br_7_]^2+^ molecule (**entry 31**), which contains a bulky 1,3‐di(2,4,6‐trimethylbenzyl)benzimidazol‐2‐ylidene (^MesCH2^bimy) group (Figure 4k) as the NHC ligand, was also synthesized by Crudden et al. (Figure 15b,c).^[10ab]^ In the case of **entry 31**, the ticosahedral Au_13_ are twisted by 10° to give *D*_5_ symmetry. The same group synthesized [Au_25_(^MesCH2^bimy)_10_Br_8_]^+^ (**entry 32**), in which not five but six Br are coordinated to the metal at the M_L1_ site.^[10ab]^ In this case, the L_1_ ligands include four μ‐Br and two terminal Br and this is the second‐ever example of a superatomic molecule having a terminal halide coordinated to the M_L1_ site, following **entry 10** ([Ag_17_Au_8_(PPh_3_)_10_Cl_10_]^0^). The number of valence electrons in **entries 31** and **32** was estimated to be 16, indicating that a closed shell structure was formed in conjunction with the NHC ligands.^[10ab]^ The **entry 31** exhibited PL with a higher Φ_PL_ value of 15% compared with other Au‐based superatomic molecules. In this molecule, the rigidity of the ligand layer was increased as a result of CH–*π* or *π*–*π* interactions between the surface ligands, resulting in a higher Φ_PL_.^[10ab]^

### 2.5 M_25_ Protected by Metal Complexes and Other Ligands

**Figure 16:**
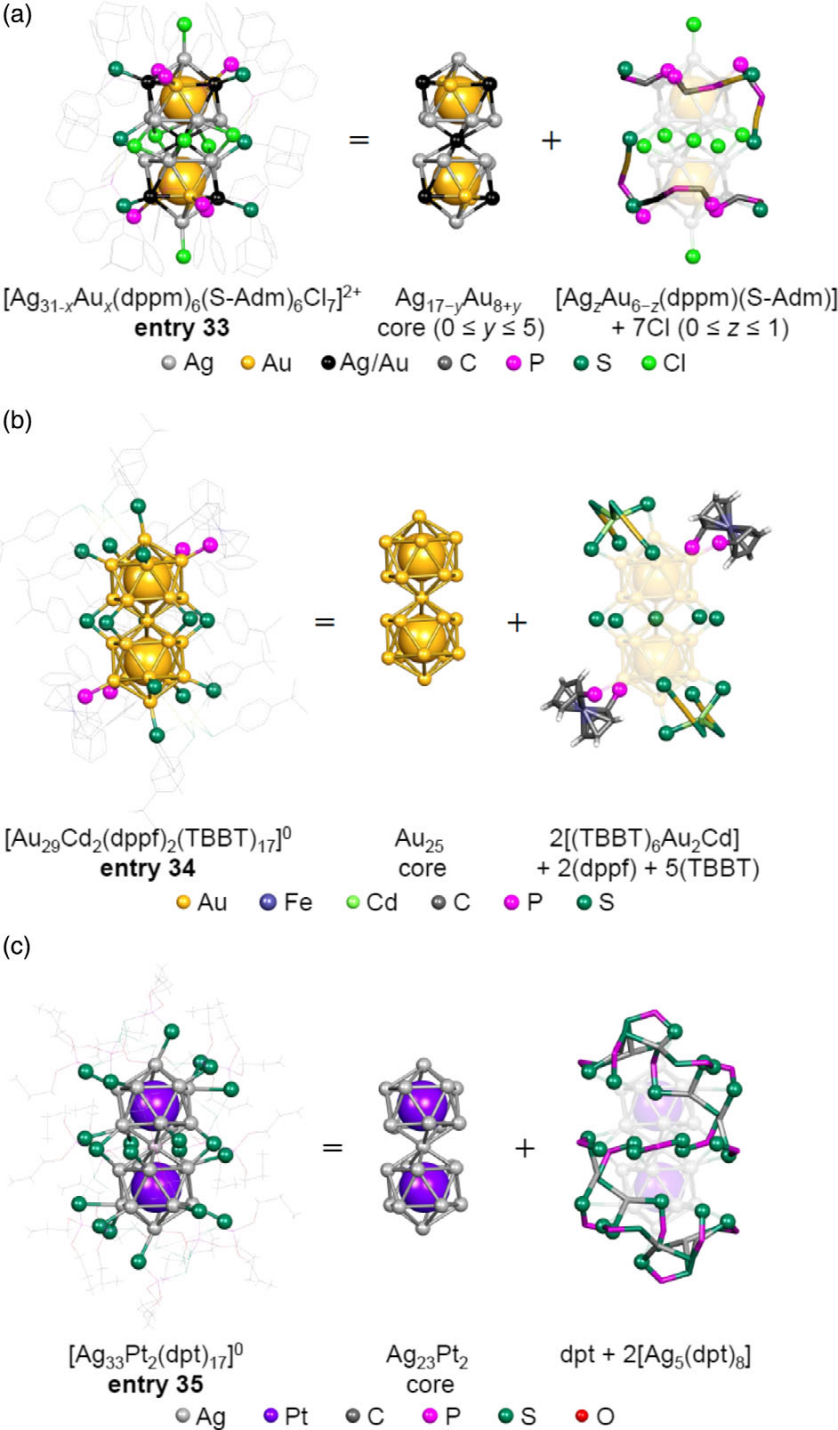
Geometrical structures and the anatomies of: a) [Ag_31−*x*_Au_*x*_(dppm)_6_(S‐Adm)_6_Cl_7_]^2+^ (**entry 33**),^[10h]^ b) [Au_29_Cd_2_(dppf)_2_(TBBT)_17_]^0^ (**entry 34**),^[10ac]^ and c) [Ag_33_Pt_2_(dpt)_17_]^0^ (**entry 35**).^[10ad]^

Zhu et al. reported the synthesis of [Ag_31−*x*_Au_*x*_(dppm)_6_(S‐Adm)_6_Cl_7_]^2+^ (S‐Adm = 1‐adamantanethiolate) molecules (Figure 4n; **entry 33**) with varying numbers of Au atoms (**Figure** **16**a).^[10h]^ These superatomic molecules comprised a metal complex composed of metal‐diphosphine‐SR ligands around an M_25_ core made of Au and Ag. In the case of **entry 33**, the molecule was stable in solution for 20 days.^[10h]^ The same group also reported the synthesis of another superatomic molecule with the formula [Au_29_Cd_2_(dppf)_2_(TBBT)_17_]^0^ (dppf = 1,1'‐bis(diphenylphosphino)ferrocene (Figure 4i), TBBT = 4‐*tert*‐butylbenzenethiolate (Figure 4p; **entry 34**)) (Figure 16b).^[10ac]^ In [Au_29_Cd_2_(dppf)_2_(TBBT)_17_]^0^, five μ‐S were coordinated to the M_L1_ site of the Au_25_ core while the M_L2_ and M_L3_ coordination sites were associated with a (TBBT)_6_Au_2_Cd complex having four monodentate S atoms and a ferrocene‐bridged diphosphine.^[10ac]^ These two metal clusters had a more complex geometry than those described in Section 2.1, 2.4 although the number of valence electrons in these structures was estimated to be 16, similar to other Au_25_ superatomic molecules. Liu et al. also reported the synthesis of a metal cluster with the formula [Ag_33_Pt_2_(dpt)_17_]^0^ (dpt = dipropyl dithiophosphate (Figure 4o; **entry 35**)) (Figure 16c). A SC‐XRD analysis established that an Ag−dpt network surrounded the Ag_23_Pt_2_ core in this structure.^[10ad]^

These M_25_ superatomic molecules incorporating metal complexes may exhibit physicochemical properties different from those of M_25_ superatomic molecules protected only by ligands, because the ligand layers in the former contain metal atoms. Therefore, it is expected that future studies of these superatomic molecules will focus on not only the stability but also catalytic activity of these materials.

### 2.6 Longer Linear M_12*n*+1_ Superatomic Molecules (*n* ≥ 3)

**Figure 17:**
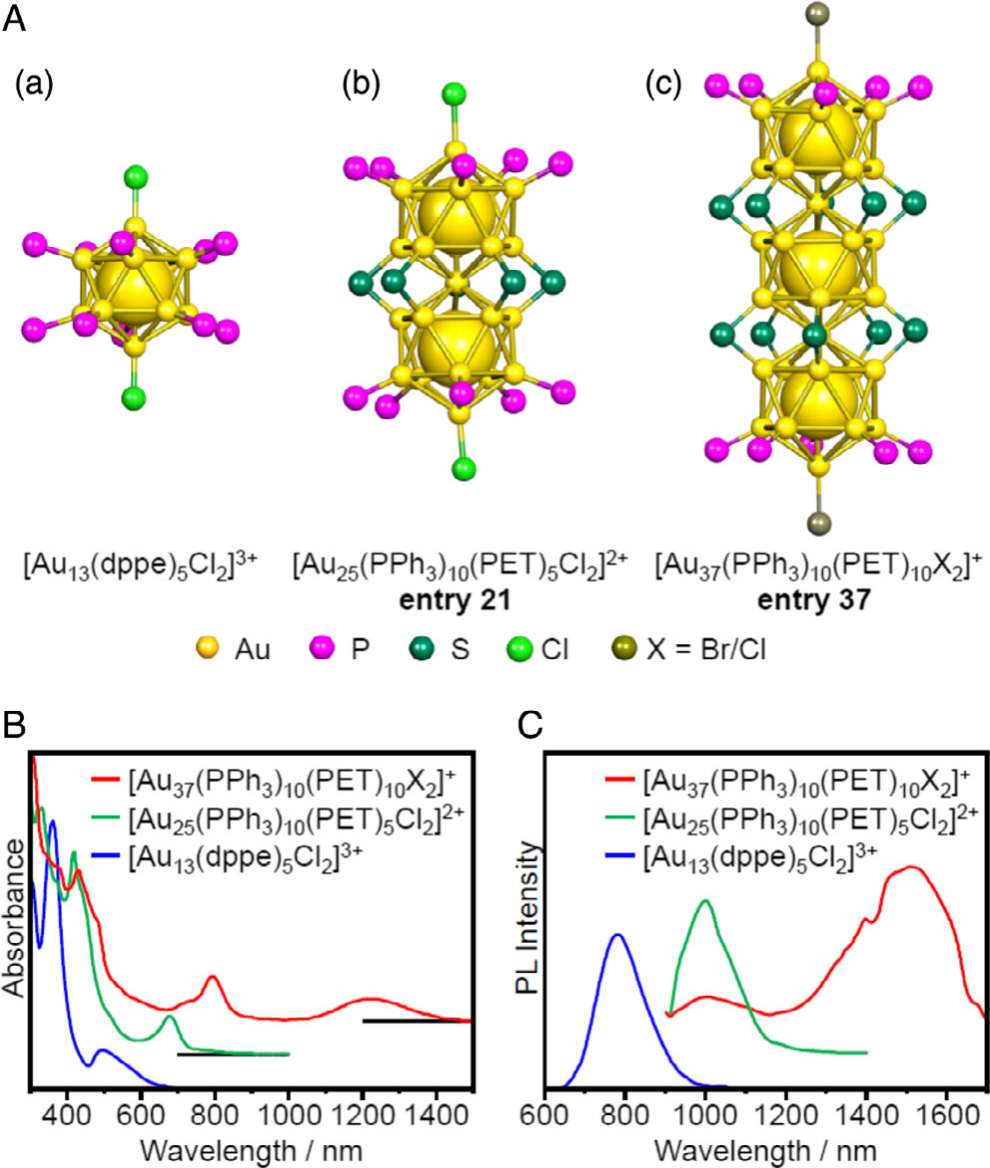
A) Framework structures of: a) the [Au_13_(dppe)_5_Cl_2_]^3+^ superatom,^[5a]^ b) the [Au_25_(PPh_3_)_10_(PET)_5_Cl_2_]^2+^ superatomic molecule (entry 21),^[10t]^ and c) the [Au_37_(PPh_3_)_10_(PET)_10_X_2_]^+^ superatomic molecule (entry 37).^[10t]^ B) UV–vis–NIR absorption and C) PL spectra of these compounds. Note that each PL spectra was recorded on a different spectrometer and so these spectra cannot be directly compared. Data in (B) and (C) are replotted from refs. [10ae] (red lines), [23] (green lines), and [27] (blue lines).

Organic dyes such as cyanines and acenes comprise 1D conjugated systems and the absorption of these materials at longer wavelengths tends to be red‐shifted as the conjugation is extended.^[^ ^33^ ^]^ Similarly, in the case of vertex‐sharing 1D M_12*n*+1_ superatomic molecules, a shift to longer wavelengths occurs in conjunction with increments in the number of the connection. Jin et al. synthesized [Au_37_(PPh_3_)_10_(PET)_10_X_2_]^+^ (X = Br or Cl; **entry 37**) (**Figure** **17**A), in which three Au_13_ units were linearly connected through the sharing of a vertex Au atom.^[10ae]^ These molecules were found to have geometries similar to that of [Au_37_(PH_3_)_10_(SCH_3_)_10_Cl_2_]^+^ (PH_3_ = phosphine, SCH_3_ = methanethiolate)^[^ ^34^ ^]^ which had been theoretically predicted by Nobusada et al. 7 years prior. The [Au_37_(PR_3_)_10_(SR)_10_X_2_]^+^ structure had 24 valence electrons and the [Au_37_]^13+^ core was isolobal to Ne_3_.^[^ ^35^ ^]^ As shown in Figure 17B, the peaks at longer wavelengths of [Au_13_(dppe)_5_Cl_2_]^3+^, [Au_25_(PPh_3_)_10_(PET)_5_Cl_2_]^2+^, and [Au_37_(PPh_3_)_10_(PET)_10_X_2_]^+^ were red‐shifted as the number of Au_13_ icosahedra were increased. Consequently, absorption was obtained up to approximately 1,200 nm in the spectrum acquired from the [Au_37_(PPh_3_)_10_(PET)_10_X_2_]^+^.^[10ae]^ These linearly connected superatoms have also shown red‐shifted PL emission wavelengths (Figure 17C).^[^ ^23^, ^27^ ^]^

**Figure 18:**
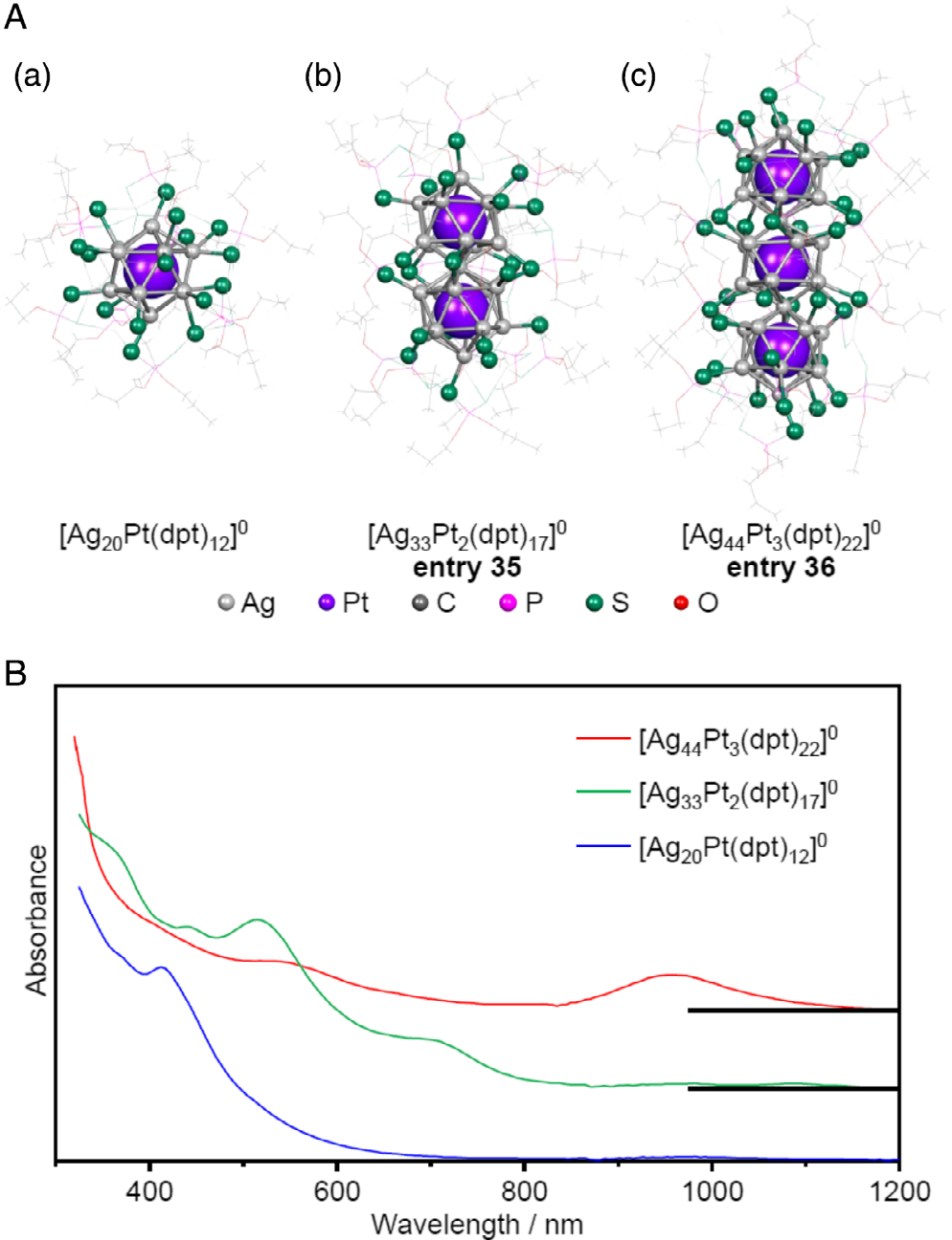
A) The geometrical structures of: a) [Ag_20_Pt(dpt)_12_]^0^, b) [Ag_33_Pt_2_(dpt)_17_]^0^ (**entry 35**), and c) [Ag_44_Pt_3_(dpt)_22_] (**entry 36**).^[10ad]^ B) The UV–vis–NIR absorption spectra of [Ag_20_Pt(dpt)_12_]^0^, [Ag_33_Pt_2_(dpt)_17_]^0^ (**entry 35**), and [Ag_44_Pt_3_(dpt)_22_] (**entry 36**). B) Data in (B) are replotted from ref. [10af].

In the case of [Au_37_(PPh_3_)_10_(PET)_10_X_2_]^+^, the linear M_37_ core is covered only by simple ligands. However, there have recently been several reports of superatomic molecules incorporating metal complexes that surround a 1D M_12*n*+1_ (*n* ≥ 3) core. As an example, Liu et al. produced [Ag_44_Pt_3_(dpt)_22_]^0^ (**entry 36**), in which an Ag_10_(dpt)_22_ shell surrounds a linear Ag_34_Pt_3_ core formed by the vertex sharing of three Ag_12_Pt groups (**Figure** **18**A).^[10ad]^ This metal cluster is considered part of a series of superatomic families comprising [Ag_20_Pt(dpt)_12_]^0^ and [Ag_33_Pt_2_(dpt)_17_]^0^ (**entry 35**) (Figure 18A). The number of valence electrons in [Ag_44_Pt_3_(dpt)_22_]^0^ was calculated to be 22 and the [Ag_34_Pt_3_]^12+^ core of this molecule was isolobal to [I_3_]^−^.[^10^, ^35^] Comparing the absorption spectra of [Ag_20_Pt(dpt)_12_]^0^, [Ag_33_Pt_2_(dpt)_17_]^0^ and [Ag_44_Pt_3_(dpt)_22_]^0^ indicates that absorption at long wavelengths was redshifted with increasing the number of the connection (Figure 18B), as shown in Figure 18A.^[10ad]^

**Figure 19:**
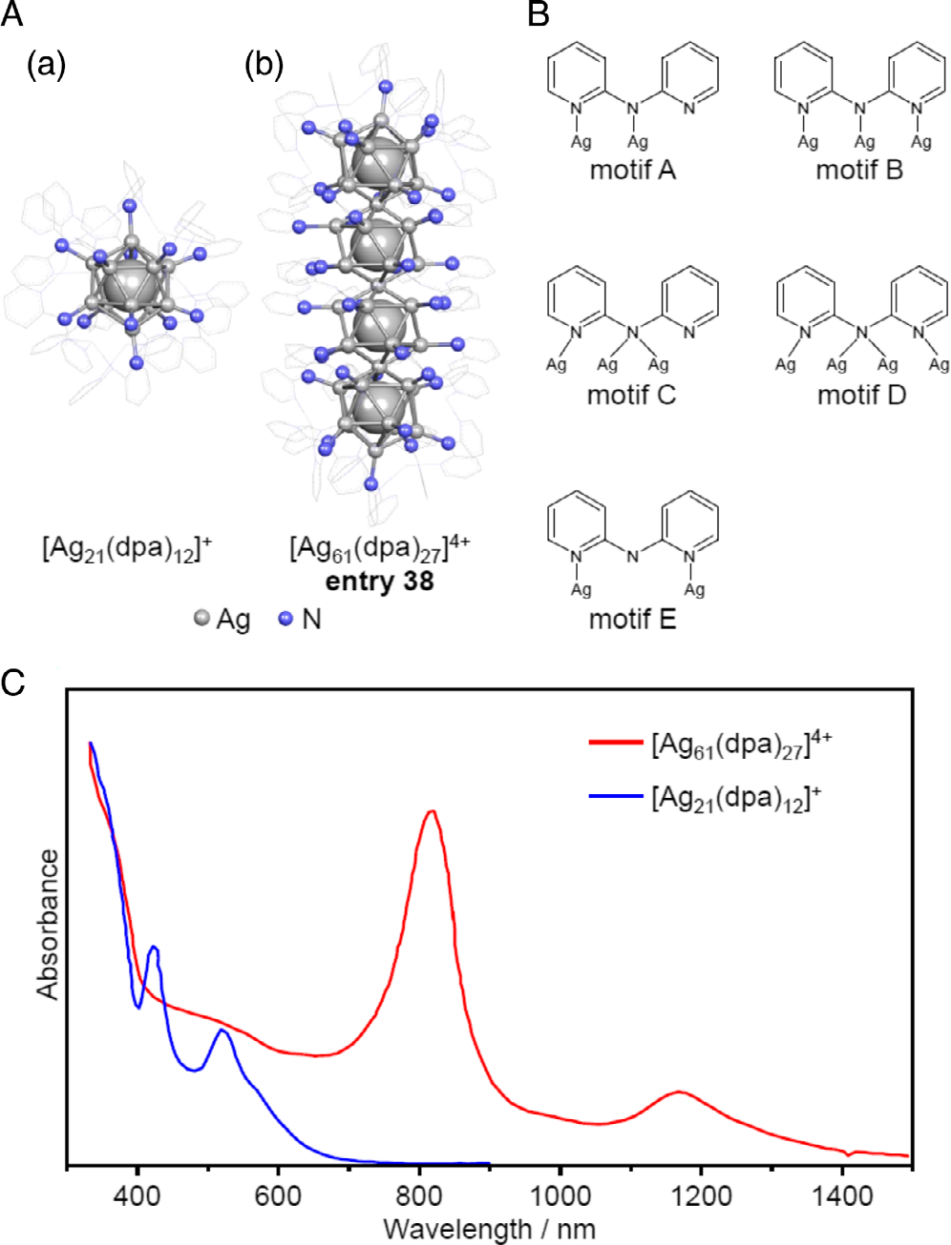
A) Geometrical structures of: a) the [Ag_21_(dpa)_12_]^+^ superatom^[^ ^36^ ^]^ and b) the [Ag_61_(dpa)_27_]^4+^ superatomic molecule (entry 38).^[10af]^ B) The binding motifs of the dpa ligands. Reproduced with permission.^[10af]^ Copyright 2021, American Chemical Society. C) The UV–vis–NIR absorption spectra of [Ag_21_(dpa)_12_]^+^ and [Ag_61_(dpa)_27_]^4+^. Data in (C) are replotted from ref. [10af].

Wang et al. synthesized [Ag_61_(dpa)_27_]^4+^ (dpa = dipyridylaminide (Figure 4q; **entry 38**), which had a linear Ag_49_ core formed by four Ag_13_ (**Figure** **19**A).^[10af]^ This superatomic molecule can be considered as a connected structure made from the [Ag_21_(dpa)_12_]^+^ superatoms previously reported by the same group.^[^ ^36^ ^]^ The dpa ligand has three N atoms and so these ligands had various coordination forms when incorporated into the [Ag_21_(dpa)_12_]^+^ and [Ag_61_(dpa)_27_]^4+^ molecules (Figure 19B). The number of valence electrons in [Ag_61_(dpa)_27_]^4+^ was estimated to be 30 and the electronic structure of the [Ag_49_]^19+^ core of this structure was equivalent to that of [I_4_]^2−^.^[10af]^ The Ag_49_ core of [Ag_61_(dpa)_27_]^4+^ was determined to have a length of 2.11 nm along its long axis, equivalent to the distance of approximately 2 nm over which localized surface plasmon resonance occurs.^[^ ^37^ ^]^ However, [Ag_61_(dpa)_27_]^4+^ has a molecular‐like electronic structure and the absorption peak at 1,170 nm in the optical absorption spectrum of this material (Figure 19C) is not attributed to plasmons but rather to an electronic transition from the HOMO to LUMO, whose electron distributions are localized in the Ag_49_ core.^[10af]^ The peak at 819 nm (with a molar absorption coefficient of *ε* = 6.2 × 10^4^ m ^−1^ cm^−1^) was attributed to the ligand to metal charge transfer (LMCT) from a motif to the core, similar to the peak at 512 nm (*ε* = 2.0 × 10^4^ m ^−1^ cm^−1^) in the spectrum of [Ag_21_(dpa)_12_]^+^.^[10af]^ Figure 19C shows that the oscillator strength of these LMCT peaks was enhanced by a factor of approximately 3 in the case of multiple connections.^[10af]^

As noted, the optical absorbance at longer wavelengths exhibited by linear superatomic molecules is primarily the result of transitions from the orbitals close to the HOMO to the orbitals close to LUMO, which are symmetrical to the fivefold symmetric long axis of the molecule. As an example, [Au_25_(PPh_3_)_10_(SR)_5_Cl_2_]^2+^ (**entries 20–22**) and [Au_37_(PPh_3_)_10_(PET)_5_X_2_]^+^ (**entry 37**), both of which have *D*_5h_ symmetry, absorb at longer wavelengths because of an orbital transition from the occupied *a*_2_”(Σ) to the virtual *a*_1_´(Σ*). As the number of connections increases, the energy gap between these orbitals decreases, resulting in redshifts of the absorption peaks of these species.[^10^, ^24^, ^34^] In the case of [Ag_61_(dpa)_27_]^4+^ (**entry 38**), the absorption peak at 1,200 nm originated from the transition between orbitals having nodes vertical to the long axis (six nodes → seven nodes), corresponding to an orbital transition from the occupied *a*_1 × g_ to the virtual *a*_2u_ in an [Ag_49_]^19+^ core, which has *D*_5d_ symmetry.^[10af]^ On this basis, similar superatomic molecules consisting of five or more superatoms should exhibit similar absorption characteristics and would be expected to absorb at longer wavelengths compared with molecules comprising four linearly connected superatoms.

Even organic dyes with linearly connected conjugated systems show narrowing of the energy gaps between orbitals as the number of connections increases. However, it is difficult to obtain infrared fluorescence from these compounds with high quantum yields because of the energy gap law^[^ ^23^, ^38^ ^]^ and the photobleaching effect associated with the instability of the photoexcited states.^[^ ^39^ ^]^ In contrast, superatoms and superatomic molecules composed of metal atoms are highly stable in response to light exposure and so no significant destabilization of the excited state occurs even as the number of connections is increased. In the future, it is expected that a deeper understanding of the excited states of linear superatomic molecules will be obtained,[^13^, ^14^, ^40^] and thereby the research on their application as infrared fluorescent materials will become more active than at present.

## 3 Conclusion and Perspectives

This review summarized the geometries and optical properties of linear M_12*n*+1_ superatomic molecules (*n* ≥ 2) formed by the vertex sharing of M_13_ superatoms. The following key points are emphasized:

### 3.1 Length of the M_12*n*+1_ Core

Linear M_12*n*+1_ superatomic molecules with *n* = 2–4 have been reported to date.

### 3.2 Appropriate Ligand Combinations

Specific ligand combinations have been found to produce linear M_12*n*+1_ superatomic molecules. The ligands employed thus far comprise PR_3_ and X, PR_3_, ER and X, PR_3_, C ≡ CR and X, NHC and X, SR, metal complexes and X, SR and metal complexes, and metal complexes alone.

### 3.3 Number of Valence Electrons in Synthesizable M_12*n*+1_ Cores

M_25_ superatomic molecules that have been isolated have typically had 16 valence electrons. In this case, the electronic structure is equivalent to that of the Ne_2_ molecule (1S^2^1P^6^). However, for Au_25_(PPh_3_)_10_(SePh)_5_Cl_2_, molecules having either 16 or 17 valence electrons can be produced. For linear M_37_ superatomic molecules, those with 24 or 22 valence electrons have been reported. The former had an electronic structure equivalent to Ne_3_ and the latter to [I_3_]^−^. M_49_ superatomic molecules with 30 valence electrons have been synthesized. These molecules had an electronic structure equivalent to that of [I_4_]^2−^.

### 3.4 Stability

The stability of superatomic molecules can be enhanced by selecting the second to sixth ligand combinations noted in point (b) above, rather than the first combination. Stability can also be improved by strengthening the M_12*n*+1_ core through heteroatom substitution.

### 3.5 Optical Absorption

Light absorption at long wavelengths by these materials can be attributed primarily to transitions from orbitals close to the HOMO to orbitals close to the LUMO, both of which are symmetric to the long axis of the molecule, which has fivefold symmetry. As the number of connections increases, the energy gap between these orbitals decreases, resulting in a red shift in optical absorption. In addition, increasing the number of connections enhances the oscillator strength associated with the LMCT.

### 3.6 PL Properties

M_12*n*+1_ superatomic molecules (*n* ≥ 2) exhibit PL and the PL emission wavelengths of these molecules have been shown to undergo a redshift with increases in the number of the connection. In the case of [Au_25_(PPh_3_)_10_(SR)_5_Cl_2_]^2+^, higher Φ_PL_ and longer τ_PL_ values have been observed compared with those for other secondary near‐infrared chromophores, suggesting the potential for practical applications. Increasing the rigidity of the ligand layer via interactions between surface ligands can further enhance Φ_PL_.

### 3.7 Magnetic Properties

The [Au_25_(PPh_3_)_10_(SePh)_5_Cl_2_]^2+/+^ molecule has magnetic properties that can be controlled by varying the charge state (2+ or +).

### 3.8 Catalytic Activities

The catalytic activity of M_12*n*+1_ superatomic molecules (*n* ≥ 2) depends on the protective ligand. The reported catalytic activities include oxidation of organic molecules, catalysis of terminal alkynes to alkenes, the cyclic isomerization of alkynyl amines to indoles, and the electrochemical reduction of inorganic compound.

As described in the Introduction, it is expected that the assembly of superatomic molecules from superatoms will lead to the creation of new materials with unique physical properties and functions that are different from those of existing substances. As an example, the chirality resulting from the structures of certain superatomic molecules cannot be obtained in molecules having isoelectronic structures. The creation of stable and highly efficient near‐infrared luminescent materials by increasing the number of the connection of superatomic molecules is another unique phenomenon. It is expected that many M_12*n*+1_ superatomic molecules having novel structures and physicochemical properties will be created in the future by making good use of the above information.

Although this review focused solely on linear connections of superatomic molecules, ring formations are also possible.[^10^, ^41^] In superatomic molecules, as is the case for more typical molecules, both the electronic structure and physicochemical properties are changed as a consequence of ring formation.^[41c]^ Although there have been only a few reports to date concerning cyclic superatomic molecules, it is expected that many studies will be conducted in the future.

## Conflict of Interest

The authors declare no conflict of interest.
